# Immune regulatory adjuvant approach to mitigate subcutaneous immunogenicity of monoclonal antibodies

**DOI:** 10.3389/fimmu.2024.1496169

**Published:** 2024-12-10

**Authors:** Nicole L. Jarvi, Manali Patel, Krithika A. Shetty, Nhan H. Nguyen, Brooke F. Grasperge, Donald E. Mager, Robert M. Straubinger, Sathy V. Balu-Iyer

**Affiliations:** ^1^ Department of Pharmaceutical Sciences, University at Buffalo, The State University of New York, Buffalo, NY, United States; ^2^ Truvai Biosciences, LLC, Buffalo, NY, United States; ^3^ Division of Veterinary Medicine, Tulane National Primate Research Center, Covington, LA, United States; ^4^ Enhanced Pharmacodynamics, LLC, Buffalo, NY, United States

**Keywords:** protein therapeutics, immunogenicity, immune tolerance, anti-drug antibodies, subcutaneous administration, formulation

## Abstract

**Introduction:**

Immunogenicity continues to be a challenge for development and clinical utility of monoclonal antibodies, and there are gaps in our current ability to prevent anti-drug antibody development in a safe and antigen-specific manner.

**Methods:**

To mitigate immunogenicity of monoclonal antibodies administered subcutaneously, O-phospho-L-serine (OPLS)—the head group of the tolerance-inducing phospholipid, phosphatidylserine—was investigated as an immunoregulatory adjuvant.

**Results:**

Formulations of adalimumab, trastuzumab or rituximab with OPLS showed reduction in relative immunogenicity in mice compared to vehicle formulations, indicated by reduced anti-drug antibody development and significant reductions in CD138+ plasma cell differentiation in bone marrow. Titer development toward recombinant human hyaluronidase, a dispersion enhancer that was co-formulated with monoclonal antibodies, was similarly reduced. Subcutaneous administration of adalimumab with OPLS resulted in a two-fold increase in expression of type 1 regulatory (Tr1) T cell subset in the spleen. This is consistent with in vitro studies where co-culturing of dendritic cells primed with ovalbumin in the presence and absence of OPLS and antigen specific T-cells induced expression of Tr1 phenotype on live CD4+ T cells.

**Conclusion:**

This adjuvant does not impact immune competence of non-human primates and mice, and repeated administration of the adjuvant does not show renal or hepatic toxicity. Formulation of monoclonal antibodies with the immunoregulatory adjuvant, OPLS, was found to be safe and effective at mitigating immunogenicity.

## 1 Introduction

The subcutaneous (SC) route of administration has been increasingly considered for delivery of protein therapeutics and monoclonal antibodies, although it has a risk for enhanced immunogenic potential compared to traditional intravenous (IV) delivery. SC delivery is convenient and more cost-effective, but monoclonal antibody (mAb) bioavailability can vary, and the SC extracellular matrix introduces barriers to protein dispersion and absorption (1). Furthermore, immunogenicity incidence of mAbs is sometimes elevated in the SC treatment arm compared to IV treatment (2). For example, no patients receiving IV trastuzumab developed anti-drug antibodies (ADA) (n=12), however 14% (8/58) of patients receiving SC trastuzumab were positive for ADA (3). To mitigate immunogenicity, clinical strategies employ general immune suppressants, such as prednisone, methotrexate, bortezomib, or cyclophosphamide (4–7). However, immune suppression can increase the risk of secondary infections, and reduction in immune activation is undesirable for concomitant cancer therapies that rely on immune cell mediated effector functions. In this case, an antigen-specific approach that is safe and effective is required in order to prevent immunogenicity of the therapeutics while maintaining general immune system activity.

Phosphatidylserine (PS)—a naturally occurring bioactive phospholipid has been shown to induce tolerogenic immune responses against therapeutic proteins (8–12). Antigen-specificity of PS-mediated tolerance induction has been demonstrated in multiple studies where mice remain hyporesponsive to the tolerized antigen but mount a robust immune response to an irrelevant antigen, such as ovalbumin (OVA) (10, 13). This tolerogenic activity is proposed to signal through PS receptors on dendritic cells (DCs), such as TIM-4, and O-phospho-L-serine (OPLS), which is the head group of PS containing the critical structural requirements for receptor binding (14–17). It has been established by our group that OPLS is a multi-functional excipient capable of improving physical stability and reducing immunogenicity of therapeutic proteins (12, 18–20). For example, Pompe mice treated with recombinant acid alpha glucosidase (GAA) in the presence of OPLS showed a reduction in anti-GAA antibody titers, and when these mice were rechallenged with GAA alone, they continued to show hypo-responsiveness indicating the immune regulatory properties of OPLS (12). Furthermore, mouse bone marrow-derived dendritic cells (BMDC) primed with Factor VIII (FVIII) in the presence of OPLS were less able to promote a robust anti-FVIII immune response when transferred into naïve Hemophilia A mice, compared to FVIII-primed BMDCs (21).

In this study, the ability of OPLS to reduce the relative immunogenicity of mAbs—adalimumab (ADM), trastuzumab (TTZ), and rituximab (RTX) —as well as hyaluronidase was investigated when co-administered subcutaneously. ADM as a fully human IgG1 antibody was presumed to have no immunogenicity implication, but the surge of ADM biosimilars in Europe and U.S. in 2023 underscores this challenge evidenced by comparable and moderate immunogenicity in recent trials (22). MAbs for SC administration can be formulated with recombinant human hyaluronidase (rHuPH20) to improve absorption and bioavailability (23). For example, RTX (Rituxan Hycela^®^) and TTZ (Herceptin Hylecta^®^) were formulated for SC administration with 2000 U/mL of rHuPH20 to facilitate the delivery of volumes greater than 3 mL (24). Other examples of biologics in various stages of development for SC delivery with rHuPH20 are efgartigimod (FcRn targeting antibody fragment), nivolumab (PD-1 targeting antibody), ocrelizumab (CD20 targeting antibody), and atezolizumab (PD-L1 targeting antibody) (25–28). rHuPH20 acts as a dispersion enhancer by catalyzing the breakdown of hyaluronic acid in a local, transient manner to improve protein movement through the interstitium. Although rHuPH20 is a recombinant protein based on a naturally occurring human testicular hyaluronidase (PH20), clinical immunogenicity is observed as pre-existing and treatment-emergent or -boosted anti-rHuPH20 antibodies (29, 30). Both package inserts for Herceptin Hylecta^®^ and Rituxan Hycela^®^ state that the clinical relevance of ADA, whether directed against the antibody or rHuPH20, is unknown (31, 32).

Here, RTX and TTZ were co-formulated with rHuPH20 to mimic clinical preparations of Rituxan Hycela^®^ and Herceptin Hylecta^®^, respectively, and anti-RTX and anti-TTZ antibody responses were followed in an addition to anti-rHuPH20 antibody responses. In the presence of OPLS, SC injection of RTX or TTZ resulted in reduced development of ADA, decreased differentiation of long-lived plasma cells, and limited activation of CD4^+^ T cells. Formulation of ADM with OPLS also reduced anti-ADM antibody development, and an increase in splenic regulatory type 1 T cells (Tr1) was observed. Furthermore, in two species, short-term, repeat-dose toxicity studies, concentrations of OPLS ranging up to 450 mM did not impact the general state of immunocompetence of the animals. OPLS also produced no evidence of renal, hepatic, or injection site damage, suggesting a wide margin of safety around its anticipated therapeutic concentration (50 mM). Results suggest that the addition of OPLS into a formulation designed for SC administration can reduce the risk of ADA development.

## 2 Materials and methods

### 2.1 Materials

Biosimilar research-grade rituximab, trastuzumab, and adalimumab were purchased from Bio X Cell (Lebanon, NH). EndoGrade ovalbumin was purchased from BioVendor (Asheville, NC). OPLS, keyhole limpet hemocyanin (KLH), cyclophosphamide monohydrate (CYP), polysorbate 20, Collagenase D, DNAse I, TMB substrate, HRP conjugated anti-mouse IgM and IgG antibodies, vitamin D3 (Vit D3), dexamethasone (Dex), DMSO, 2-mercaptoethanol, and buffer reagents (L-histidine, L-histidine hydrochloride monohydrate, L-methionine, α,α-trehalose dihydrate, edetate sodium, calcium chloride, and dibasic sodium phosphate) were purchased from Sigma Aldrich (St. Louis, MO). Recombinant human hyaluronidase (HYLENEX^®^ NDC 18657011704) was purchased from McKesson (Irving, TX) through the Laboratory Animal Facilities at the University at Buffalo. Anti-RTX (clone 6C1), anti-ADM (clone 3C2), and anti-TTZ antibody standards (clone 11C4) were purchased from GenScript (Piscataway, NJ). Alkaline phosphatase conjugated anti-rhesus IgM and IgG antibodies were obtained from LifeSpan Biosciences (Seattle, WA) and Southern Biotech (Birmingham, AL). RPMI-1640, EDTA solution, HEPES solution, and heat inactivated fetal bovine serum (HI-FBS) were sourced from Corning (Corning, NY). Bovine serum albumin (30% solution) was purchased from Rockland Immunochemicals (Pottstown, PA). Fluorescently labeled antibodies and compensation beads were purchased from Tonbo Biosciences (San Diego, CA), Biolegend (San Diego, CA), or BD Biosciences (San Jose, CA). Anti-PS antibody was purchased from EMD Millipore (Billerica, MA). Creatine kinase detection reagent was obtained from Thermo Fisher Scientific (Waltham, MA).

### 2.2 Animals

Male and female Swiss Webster (SW) mice and male Cr1: CD1 (ICR) mice, all aged 4-5 weeks, were purchased from Charles River (Wilmington, MA) and housed in the Laboratory Animal Facilities at the University at Buffalo under conventional conditions. SW mice were used for relative immunogenicity studies due to ease of use and availability, and for this purpose an outbred model may be more relevant since inter-individual variability in clinical immunogenicity is significant. CD-1 mice are an outbred strain and a well-accepted animal model for general toxicology and immunotoxicology evaluations (33). Experiments were initiated one week after their arrival to facilitate acclimation. Mice were housed two per cage and provided standard food and water ad libitum. All animal care, handling, and experiments were performed in strict accordance with the principles of the Institutional Animal Care and Use Committee (IACUC) at the University at Buffalo. Animal body weight was recorded weekly. For toxicity studies in non-human primates (NHP), three male Rhesus macaques (3-4 years of age) at the Tulane National Primate Research Center (TNPRC) (Covington, LA) were used. Animals were pair-housed in commercial primate caging within an Animal Biosafety Level 2 facility and had ad libitum access to commercial primate food and fresh drinking water. All husbandry, environmental enrichment, veterinary and other procedures were performed in compliance with the Guide for the Care and Use of Laboratory Animals and the Animal Welfare Act.

### 2.3 Relative immunogenicity studies

Relative immunogenicity studies were conducted in SW mice by administering weekly SC injections of each protein formulated in buffer (vehicle) containing various concentrations of OPLS. In the first study design, groups of mice (n=8/group, 50:50 males and females) were administered four weekly SC injections of TTZ and rHuPH20 formulated in vehicle containing 0 or 25 mM OPLS or RTX and rHuPH20 formulated in vehicle containing 0, 25, or 100 mM OPLS. Although gender differences in immune responses were not expected, both male and female mice were included. The fixed therapeutic doses of RITUXAN HYCELA^®^ (1400 mg RTX and 23400 U rHuPH20) and HERCEPTIN HYLECTA^®^ (600 mg TTZ and 10000 U rHuPH20) were adjusted by weight, assuming a weight of 70 kg in humans and 25 g in mice. Mice were administered 0.5 mg RTX and 8.36 U rHuPH20 (in 160 μL) or 0.214 mg TTZ and 3.58 U rHuPH20 (in 100 μL). One week prior to the first injection, the injection site areas were shaved to observe the incidence of injection-site reactions (e.g., redness or swelling). Following the last injection, mice were given a washout period to facilitate clearance of the drug to reduce interference with ADA measurement. The 10-week washout period accommodated five half-lives of RTX and TTZ in mice (approximately 2 weeks) (34, 35). The relative immunogenicity of ADM in the presence of OPLS was tested by administering mice (n=10/group, 50:50 males and females) ADM in vehicle containing 0, 25, or 50 mM OPLS. Instead of weekly doses, one loading dose was given followed by a maintenance dose two weeks later, modeling the dosing regimen of HUMIRA^®^. The fixed loading and maintenance doses, 80 and 40 mg, respectively, were adjusted by weight following the above stated assumptions. Mice were administered SC injections of 28.5 μg ADM (in 100 μL) on week 1 and 14.3 μg ADM (in 100 μL) on week 3. A washout period of four weeks was observed to follow ADA development. In the above studies, the investigator was blinded to the treatment groups until all data acquisition and analysis was complete.

### 2.4 Toxicity studies

Dose-response relationships of OPLS with various immune, renal, hepatic, and injection site toxicity endpoints were evaluated in short-term, repeat-dose studies. Dose selection of OPLS was guided by the anticipated therapeutic formulation—50 mM OPLS. Five doses, spanning the clinically relevant dose were evaluated in mice, and the clinically anticipated dose was evaluated in NHP (rhesus macaques). The overall study design and distribution of animals among treatments groups are summarized in **Supplementary Tables 1** and **2**, respectively. Mice received daily doses of the assigned treatment (250 μL/injection) for 28 consecutive days via SC injections administered in the scruff of the neck. NHP were administered 21 daily SC doses of OPLS between the scapulae.

### 2.5 Preparation of OPLS formulations

For studies with TTZ + rHuPH20 and RTX + rHuPH20, OPLS was prepared in a buffer that approximately matched the inactive ingredients in RITUXAN HYCELA^®^: 0.53 mg/mL L-histidine, 3.47 mg/mL L-histidine hydrochloride monohydrate, 1.49 mg/mL L-methionine, and 79.45 mg/mL α,α-trehalose dihydrate in sterile water for injection, pH 5.5. The OPLS stock was adjusted to pH 5.5 using 10 M NaOH. For the ADM study, OPLS was prepared in a buffer that approximately matched that of HUMIRA^®^: 1.3 mg/mL citric acid monohydrate, 1.5 mg/mL dibasic sodium phosphate dihydrate, 12 mg/mL mannitol, 0.85 mg/mL monobasic sodium phosphate dihydrate, 6.25 mg/mL sodium chloride, and 0.3 mg/mL sodium citrate in sterile water for injection, pH 5.2. The OPLS stock was pH adjusted to pH 5.2. For BMDC cell cultures, OPLS was prepared in RPMI-1640 with 10 mM HEPES and pH adjusted to pH 7.4. For the toxicity studies, OPLS formulations were prepared in TRIS buffer: 25 mM TRIS, 150 mM or 300 mM NaCl, pH 7.0. Osmolarity of solutions was measured using a Wescor 5500 vapor pressure osmometer (Wescor Inc, Logan, UT). CYP solution (2 mg/mL) was prepared in normal saline. All OPLS formulations were 0.2-μm sterile filtered and confirmed to contain less than 0.05 EU/mL endotoxin using the LAL Endochrome-K kit from Charles River (Wilmington, MA). A phosphate assay confirmed the stock concentration of OPLS (36). OPLS formulations were stored at 4°C until use.

### 2.6 Plasma and tissue collection

In the relative immunogenicity studies for TTZ + rHuPH20 and RTX + rHuPH20, starting two weeks after the last dose, blood samples were collected from mice biweekly in heparinized capillary tubes by saphenous vein sampling. In the ADM study, saphenous blood samples were collected weekly throughout the study. At each study terminal, all mice were sacrificed by cardiac puncture to collect a terminal blood sample in acid citrate dextrose (ACD) buffer (85 mM sodium citrate, 110 mM D-glucose, 71 mM citric acid) at a 9:1 volume ratio blood: ACD. Plasma was separated by centrifugation at 5000xg for 5 min and stored at -80°C until analysis. Upon sacrifice, the inguinal lymph node from the injection draining side was collected from all mice, as well as the spleen and one femur. In the toxicity studies, non-terminal plasma and whole blood samples from mice were collected from the saphenous vein into heparin or EDTA coated capillary tubes. Terminal plasma samples from mice were collected by cardiac puncture into ACD buffer at a 7:1 volume ratio blood: ACD. Serum samples were collected by allowing whole blood with no anticoagulant to clot for 30 minutes at room temperature prior to centrifugation. EDTA- or citrate anti-coagulated plasma samples and serum samples were collected similarly from rhesus macaques. All samples were either analyzed immediately or stored at -80°C until analysis. Immediately after exsanguination, mouse spleen, liver, kidney, and injection site skin samples were harvested, weighed, and examined macroscopically.

### 2.7 Lymphocyte collection and processing

Spleen contents were released by squishing between two glass slides. Red blood cells (RBC) were lysed for 2 minutes in RBC lysis buffer (Tonbo Biosciences). Then splenocytes were washed and resuspended in MACS buffer (PBS pH 7.4, 2mM EDTA, 0.5% HI-FBS). When the draining lymph node (DLN) was isolated, as much fat as possible was removed until the tissue sample sank in a tube of cold media. DLNs were placed in digestion media (RPMI-1640, 1 mg/mL Collagenase D, 40 µg/mL DNAse I, 25 mM HEPES, and 50 µM 2-mercaptoethanol) and the DLN capsule was disrupted using two 25G needles. After shaking for 30 min at 37°C, digestion was stopped by adding EDTA to a concentration of 5 mM. Digested DLNs were gently homogenized through a 40-µm cell strainer, and cells were washed and resuspended in MACS buffer. Femurs were isolated and cleaned with 70% isopropyl alcohol and gauze. After snipping the ends of the bone to create a hollow tube, the bone marrow was flushed out using PBS and a 26G needle and syringe. Cells were passed through a 100-µm cell strainer then washed and resuspended in MACS buffer. Samples were counted on the Countess II (Invitrogen), and approximately 1 x 10^6^ live cells were added to 12x75 mm polystyrene tubes for staining.

### 2.8 Lymphocyte staining and flow cytometry

SW mouse lymphocytes were stained with Ghost dye violet 450 viability stain (Tonbo Biosciences), according to product recommendations, then incubated in 100 µL MACS buffer with 1 μL/test Fc shield (2.4G2) to reduce non-specific binding. Bone marrow cells were surface stained with anti-mouse CD138 APC-Cy7 (281-2), CD19 PE (1D3), B220 APC (RA3-6B2), and IgD FITC (11-26c.2a). DLN cells were stained with anti-mouse CD4 PerCP-Cy5.5 (GK1.5), CD3 APC (17A2), CD44 FITC (IM7), PD-1 PE (J43.1), and CTLA-4 PE-Cy7 (UC10-4F10-11). All cells were fixed in 2% buffered formalin phosphate for 30 min then washed and resuspended in MACS buffer for storage at 4°C. Where indicated, splenocytes were surface stained with anti-mouse CD4 APC-Cy7 (GK1.5), CD3 FITC (17A2), LAG-3 APC (C9B7W), CD49 PE (HMA2), and LAP PerCP-Cy5.5 (TW7-16B4) and stained intracellularly with anti-mouse FoxP3 Alexa Fluor 700 (MF-14) using a fixation/permeabilization kit (Tonbo Biosciences). Unstained and single-stain controls prepared using fresh splenocytes or compensation beads were used to set the parameter voltages and compensation matrix. 50,000 to 100,000 total cell events per sample were acquired on a BD LSRFortessa. Flow cytometry data was analyzed in FlowJo v10.7.

### 2.9 Anti-drug antibody ELISAs

Plasma samples in the relative immunogenicity studies were assayed for the presence of anti-RTX, anti-TTZ, anti-ADM, and anti-rHuPH20 IgG antibodies using in-house developed sandwich ELISAs. Standard curves of control antibody clones 6C1, 11C4, and 3C2, respectively, were used to measure concentrations (ng/mL or μg/mL) of anti-RTX, anti-TTZ, and anti-ADM IgG antibodies. Anti-rHuPH20 IgG antibodies were assayed by the Frey method (37). Detailed ELISA methods are provided in **Supplementary Material**.

### 2.10 Effect of OPLS on regulatory T cell induction *in vitro*

First, to enrich for OVA-specific T cells, SW mice were immunized subcutaneously once weekly for two weeks with 2 µg OVA in 100 µL sterile saline. Then a DC: CD4^+^ T-cell co-culture was performed. Immature BMDCs were cultured from mouse bone marrow progenitor cells as described previously and used immediately or cryopreserved in 10/90 DMSO/HI-FBS and stored in vapor phase liquid nitrogen until use (38). Immature BMDCs (1 x 10^5^ cells/mL) were plated in 24-well plates with OVA (10 µg/mL) in the presence and absence of OPLS (25 or 50 mM). Control treatments included untreated cells and Vit D3 (50 pM) + Dex (500 nM). After a 24-h incubation, CD4^+^ T cells from OVA-immunized mice were spiked into the BMDC culture for a 5:1 ratio of T cells to DCs. CD4^+^ T cells were freshly isolated from splenocytes using the Miltenyi mouse CD4^+^ T cell isolation kit. After 72 h of co-culturing, cells were stained for regulatory T cell markers and acquired on the flow cytometer as described in section 2.8. Isotype controls were prepared using Alexa Fluor 700 Rat IgG2a k (eBR2a), APC Rat IgG1 (HRPN), and PE Armenian Hamster IgG (PIP).

### 2.11 Toxicity study endpoints

A complete blood count with differential was performed for peripheral blood of mice and NHP using BC-2800 (Mindray, Mahwah, NJ) and Sysmex XT2000iV (Sysmex, Lincolnshire, IL) auto hematology analyzers, respectively. Serum chemistry markers were used to evaluate functional health of the liver and kidneys. Mouse and NHP serum samples were analyzed using a Vetscan VS2 (Abaxis diagnostics, Union City, CA) or an Olympus AU400 (Beckman-Coulter, Brea, CA) analyzer. Plasma CK concentrations were analyzed using a detection kit. Immunophenotyping was performed on lymphocytes from mouse spleens and rhesus macaque peripheral whole blood. Fc receptors on lymphocytes were blocked with anti-mouse CD16/CD32 antibody pre-treatment, followed by surface staining with anti-mouse CD3 FITC, CD4 PE-Cy7, CD8 APC, and CD19 PE. Samples were analyzed on a BD FACSCalibur flow cytometer. For NHP peripheral blood, 25 mL of an antibody cocktail containing fluorescently labelled anti-CD20, anti-CD3, anti-CD4, and anti-CD8 antibodies was added to 50 mL of EDTA-anticoagulated whole blood. RBCs were lysed, and the sample was evaluated on a BD FACSVerse flow cytometer. Anti-KLH TDAR assays have been previously optimized in CD-1 mice and Cynomolgus monkeys and were adapted in our studies (39, 40). Briefly, on study Day 15, mice were administered a single IV injection of 2 mg/animal KLH. Plasma samples collected on study Days 21 and 29 were analyzed for anti-KLH IgM and IgG titers, respectively. On study Day 8, NHP were administered a single IM injection of 10 mg/animal KLH and samples were collected on Days 18 and 22. Anti-KLH IgM and IgG antibodies were detected using a previously described ELISA (39). The presence of PS-reactive antibodies in plasma of CD-1 mice and rhesus macaques was detected using a lipid-adapted ELISA (41). Detailed ELISA methods are provided in **Supplementary Material**.

### 2.12 Microscopic tissue evaluation

Tissue specimens were fixed in 10% buffered formalin phosphate. Paraffin embedded sections (n=3/tissue/treatment group) were stained with a Hematoxylin and Eosin (H&E) stain for histological examination. Histological specimens were scored by an investigator blinded to the dosage information. Tissue sections were evaluated for the following histopathological features of tissue injury: a) inflammation, (b) fibrosis, and (c) cytopathic changes including the features of necrosis, apoptosis, cytoplasmic vacuolar change, hyperplasia, hypertrophy, atrophy, metaplasia, cell swelling, proteinaceous accumulations, fatty change, and calcification. All these features were semi-quantitatively evaluated by a single reviewer according to the following scoring system: 0 = absent; 1+ = <5% of target; 2+ = 6-25% of target; 3+ = >26% of target.

### 2.13 Statistical analysis

Statistical analysis of flow cytometry data was conducted using Student’s unpaired t-test (one-tailed) or one-way ANOVA with Dunnett’s multiple comparisons test. In ADA datasets, outliers were removed in GraphPad Prism based on the ROUT test with Q set to 0.1% (definitive) or 1% (likely) where indicated. Statistical significance was determined by Student’s unpaired t-test (one-tailed), and if the titer distribution did not pass D’Agostino & Pearson test for normality, statistical analysis was performed on log transformed titer data. Mean anti-KLH titer levels (log_2_) and immunophenotyping results in mice were compared using a one-way ANOVA with Dunnett’s multiple comparisons test. Baseline and Day 18 or Day 22 mean anti-KLH titer levels in NHP were compared using a paired two sample t-test. NHP immunophenotyping data were analyzed using a repeated measures ANOVA. Mean plasma CK concentrations in mice and NHP were compared by one-way ANOVA with Dunnett’s multiple comparisons test and a repeated measures ANOVA. P-values of less than 0.05 were considered statistically significant.

## 3 Results

### 3.1 Reduced anti-ADM antibody development in the presence of OPLS

**Figure 1:**
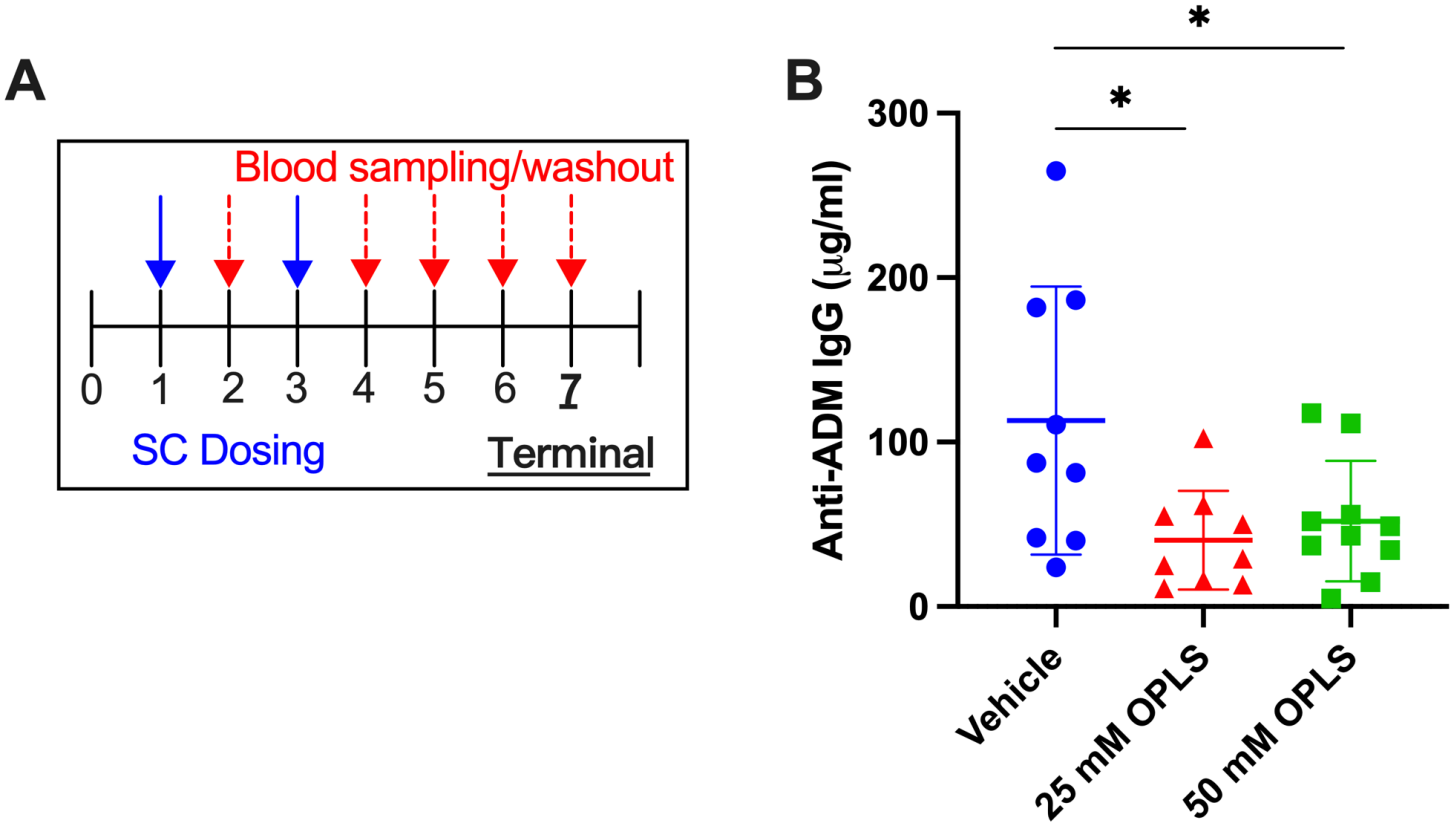
Co-formulation of ADM with OPLS reduces anti-ADM antibody formation. **(A)** Study timeline. Mice (n=10/group) were administered SC injections of ADM on week 1 (28.5 μg/mouse) and week 3 (14.3 μg/mouse) which were formulated in vehicle containing 0, 25, or 50 mM OPLS. **(B)** Anti-ADM IgG (μg/mL) in plasma at the terminal endpoint (week 7). Each dot represents an individual mouse and bars are mean ± SD. Likely outliers were removed based on the ROUT test Q=1%. Titers followed a normal distribution (D’Agostino & Pearson test). Statistical significance was tested by Student’s unpaired t-test (one-tailed). *p<0.05.

This study aimed to determine if levels of antibodies against ADM could be decreased in mice through two repeated doses of ADM with OPLS. Three groups of SW mice (n=10/group) were treated with ADM formulated in vehicle (buffer) and 25 or 50 mM OPLS. Two SC injections were given two weeks apart to simulate primary and secondary response followed by 4-week washout period to facilitate less interference from the circulating drug (**Figure 1A**). The quantitative measurement (μg/ml) of total anti-ADM IgG antibodies was assessed via in-house ELISA. At the terminal endpoint (week 7) there was a notable reduction in anti-ADM IgG antibodies in the groups receiving ADM co-formulated with 25 mM (p=0.012) and 50 mM (p=0.023) OPLS as compared to the group that received ADM in vehicle (**Figure 1B**). Mice in all treatment groups gained body weight steadily and no significant differences in body weight were observed at the end of the study (**Supplementary Figure 1**). Overall, the findings point to a potential benefit of OPLS for reducing anti-ADM antibody development.

### 3.2 OPLS co-administration reduced development of anti-TTZ and anti-rHuPH20 antibodies

**Figure 2:**
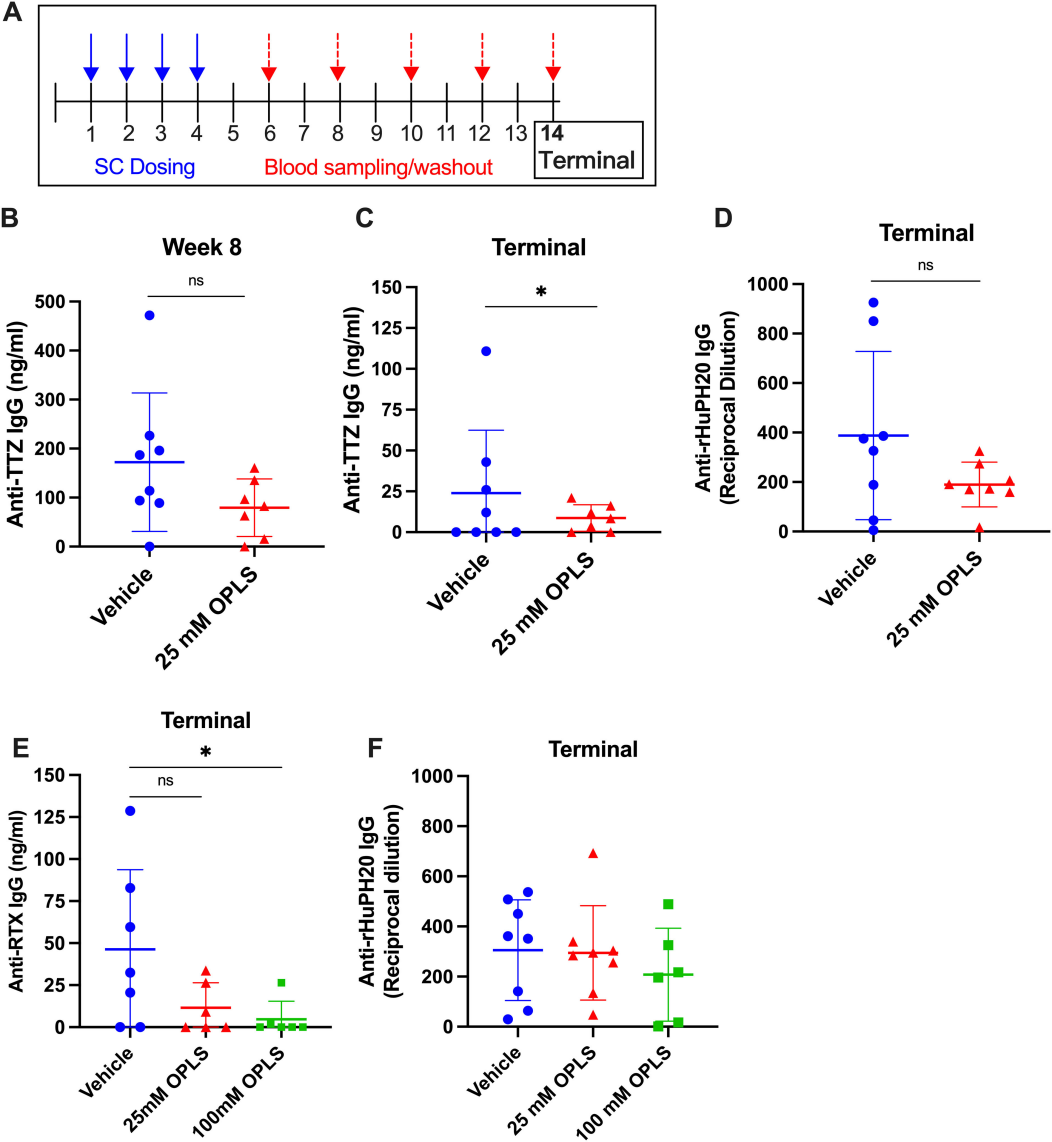
Co-formulation of OPLS with mAbs and rHuPH20 reduces ADA development. **(A)** Representative study timeline. **(B-D)** Mice (n=8/group) were administered TTZ (0.214 mg/mouse) and rHuPH20 (3.57 U/mouse) formulated in vehicle containing 0 or 25 mM OPLS once weekly for four weeks, followed by a ten-week washout period. Anti-TTZ IgG concentration (ng/mL) in plasma at **(B)** week 8 and **(C)** week 14 (terminal). **(D)** Anti-rHuPH20 IgG titers (reciprocal dilution) at the study terminal. **(E, F)** Mice (n=8/group) were administered RTX (0.5 mg/mouse) and rHuPH20 (8.36 U/mouse) formulated in vehicle containing 0, 25, or 100 mM OPLS subcutaneously once weekly for four weeks, followed by a ten-week washout period (according to **(A)**). **(E)** Anti-RTX IgG (ng/mL) in plasma at the terminal endpoint. **(F)** Anti-rHuPH20 IgG titers (reciprocal dilution) at the study terminal. In all plots, dots represent individual mice and bars are mean ± SD. In all data sets, definitive outliers were removed based on the ROUT test (Q=0.1%). Statistical significance was determined by Student’s unpaired t-test (one-tailed). When titers did not follow a normal distribution (according to D’Agostino & Pearson test), statistical analysis was performed on log-transformed data. *ns* not significant, *p<0.05.

The relative SC immunogenicity of TTZ + rHuPH20 in the presence and absence of OPLS was tested in this study. Two groups of Swiss Webster mice (n=8/group) were treated with TTZ + rHuPH20 formulated in vehicle with and without 25 mM OPLS. Weekly SC injections were given for four weeks followed by a 10-week washout period (**Figure 2A**). Anti-TTZ IgG concentration (ng/mL) in plasma was determined by an in-house ELISA. At study week 8, there was a notable reduction in anti-TTZ IgG titers in the group administered 25 mM OPLS compared to the control group receiving TTZ and rHuPH20 formulated in vehicle only (**Figure 2B**). Although anti-TTZ titers decreased across the washout period in most mice, titers remained consistently lower in the group treated with 25 mM OPLS compared to vehicle, achieving statistical significance at the terminal week (p=0.029) (**Figure 2C**). Anti-rHuPH20 titers at the terminal endpoint were trending lower in the 25 mM OPLS group compared to vehicle, but statistical significance was not achieved (**Figure 2D**). OPLS was overall well tolerated; no injection site reactions (such as redness or swelling) were observed 1 h post-injection, and no significant differences in body weight were observed between treatment groups (**Supplementary Figure 1**).

### 3.3 Dose-dependent reduction in anti-RTX antibody development upon co-formulation with OPLS

To determine the ability of OPLS to reduce the immunogenicity of RTX administered subcutaneously with rHuPH20, three groups of Swiss Webster mice (n=8/group) were treated with RTX + rHuPH20 formulated in vehicle and 25 or 100 mM OPLS. Weekly SC injections were given for four weeks followed by a 10-week washout period (**Figure 2A**). Anti-RTX IgG concentration (ng/mL) in plasma was determined by an in-house ELISA. At the terminal endpoint, the mean of anti-RTX IgG was lower in the 25 and 100 mM OPLS groups (11.5 and 4.71 ng/mL, respectively) compared to the vehicle group (46.3 ng/mL) (**Figure 2E**). High titer mice identified as definitive outliers were removed from data analysis. A statistically significant difference was observed with 100 mM OPLS group compared to vehicle group (p=0.036). Anti-rHuPH20 IgG titers trended lower in the 100 mM OPLS group compared to vehicle, but the effect was less prominent than in the TTZ + rHuPH20 study possibly due to a >2-fold higher dose of rHuPH20 (**Figure 2F**). No injection site reactions (such as redness or swelling) were observed 1 h post-injection, and no significant differences in body weight were observed between treatment groups (**Supplementary Figure 1**). The observed reduction in anti-RTX and -TTZ antibody development with OPLS may stem from a reduction in differentiation of antibody-secreting plasma cells.

### 3.4 Plasma cell differentiation is reduced in the presence of OPLS

**Figure 3:**
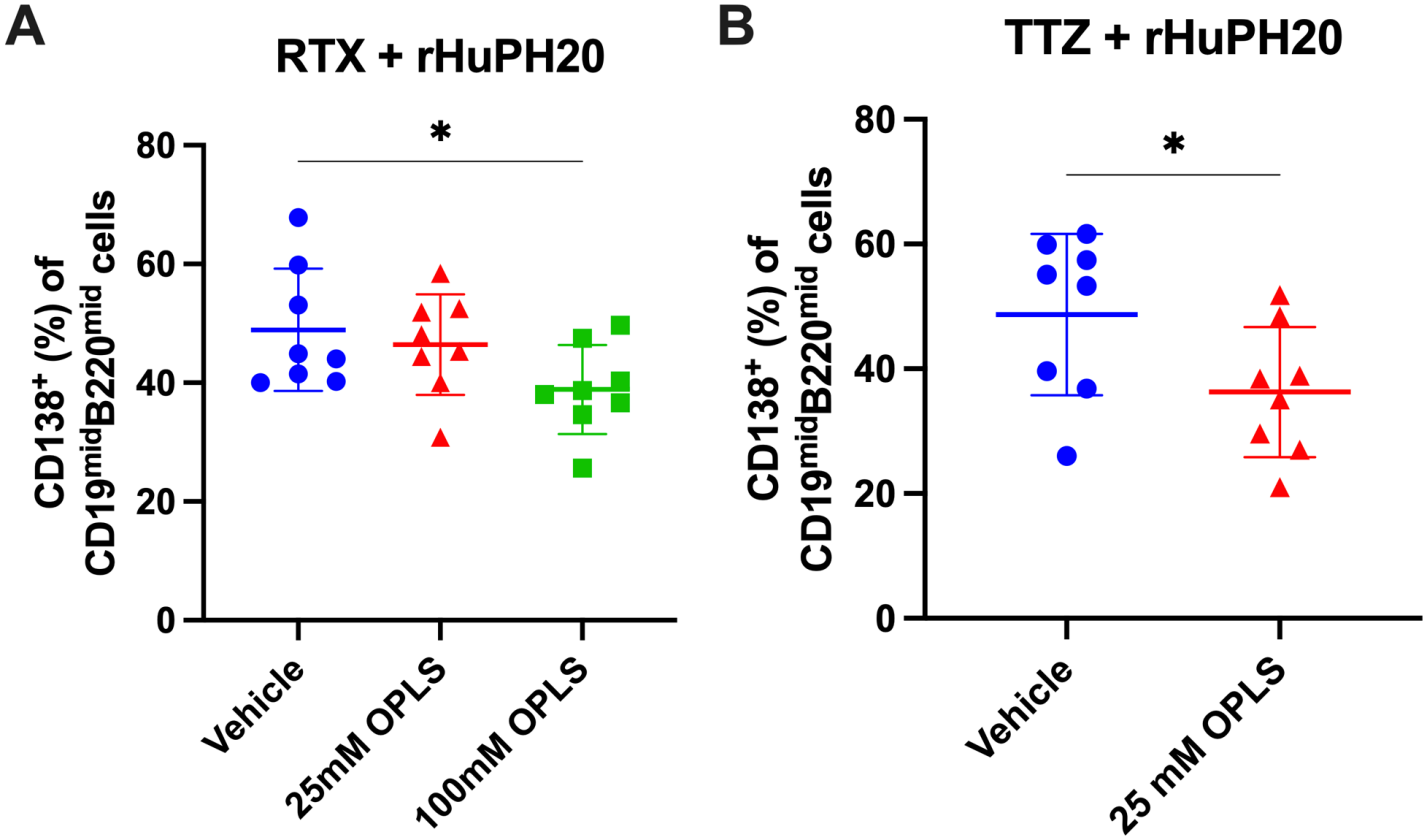
Reduction of plasma cell development in the bone marrow by OPLS co-administration with mAbs and rHuPH20. Bone marrow cells from the femur were collected at the study terminal—10 weeks past the final dose for RTX + rHuPH20 or TTZ + rHuPH20. **Supplementary Figure 2** is the flow cytometry gating strategy. Frequency (%) of CD138^+^ plasma cells out of CD19^mid^B220^mid^ cells in the bone marrow of mice treated with **(A)** RTX and rHuPH20 in vehicle, 25 mM OPLS, or 100 mM OPLS or **(B)** TTZ and rHuPH20 in vehicle or 25 mM OPLS. Dots represent individual mice and bars are mean ± SD. Statistical significance was determined by Student’s unpaired t-test (one-tailed). *p<0.05.

The frequency of long-lived plasma cells (LLPCs) was measured in the bone marrow to correlate a reduction in ADA with a reduction in plasma cell differentiation by OPLS. Plasma cells in the bone marrow were gated by CD138 expression within the cell population with low to moderate B cell marker expression (CD19 and B220) (**Supplementary Figure 2**). Mice administered RTX and rHuPH20 with 100 mM OPLS had the lowest frequency of CD138^+^ plasma cells (38.9%), and a statistically significant difference was achieved with the vehicle group (48.9%) (p=0.021) (**Figure 3A**). Similarly, mice administered TTZ and rHuPH20 with 25 mM OPLS had significantly lower CD138^+^ plasma cells (36.3%) compared to when administered in vehicle (48.7%) (p=0.026) (**Figure 3B**). Although the antigen-specificity of the plasma cells cannot be determined without performing ELISPOT analysis (42), for example, it can be reasonably assumed that significant differences are due to the study treatment. 4-week-old SW mice were randomized into blinded treatment groups and housed under identical conditions throughout the study. In correlation with reduced ADA, co-administration of mAbs with 25 to 100 mM OPLS reduced the differentiation of long-lived plasma cells.

### 3.5 T-cell activation and exhaustion markers are reduced in the presence of OPLS

**Figure 4:**
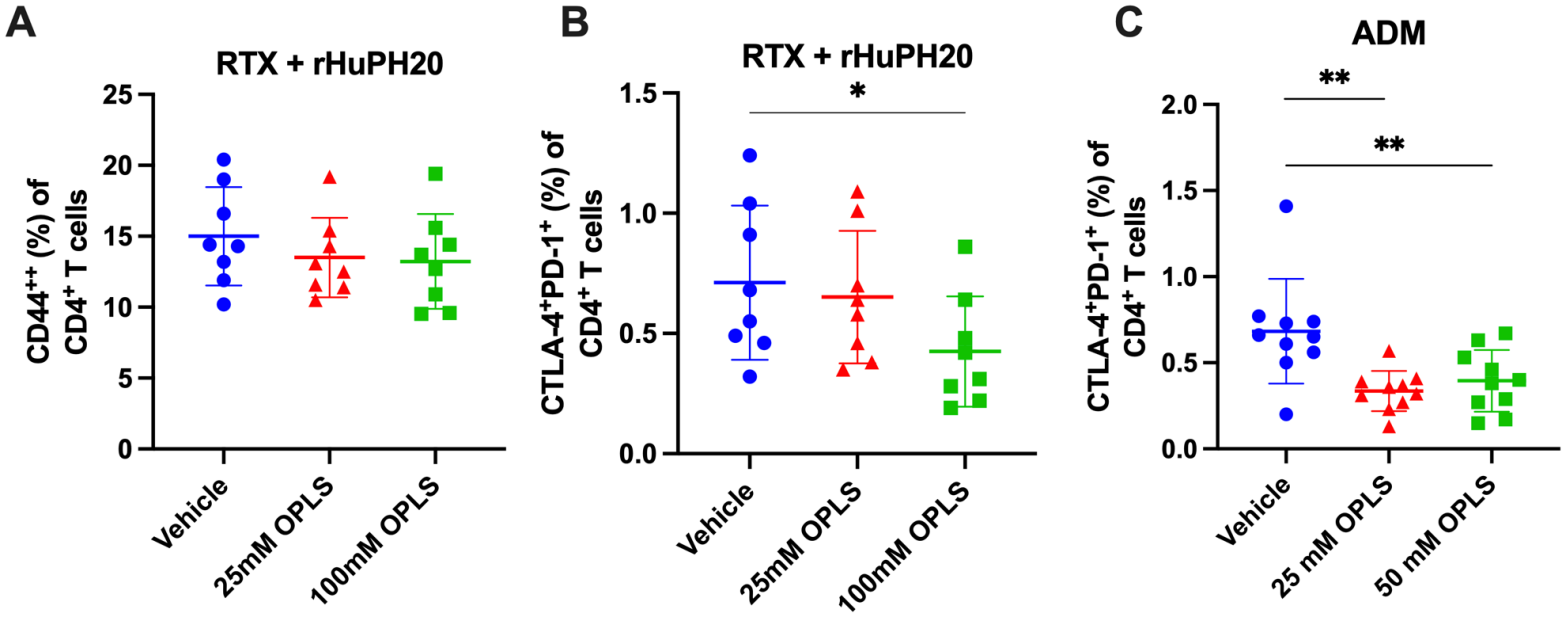
Impact of OPLS on RTX- and ADM-mediated T-cell activation when administered subcutaneously in mice. CD4^+^ T cells in the draining lymph nodes (DLN) of mice at the study terminal were stained for the activation marker CD44 and classical immune checkpoints CLTA-4 and PD-1. **Supplementary Figure 3** is the flow cytometry gating strategy. Frequency (%) of **(A)** CD44^++^ and **(B)** CTLA-4^+^PD-1^+^ cells out of live CD3^+^CD4^+^ T cells in DLN of mice administered RTX + rHuPH20 in vehicle, 25 mM OPLS, or 100 mM OPLS or **(C)** in DLN of mice administered ADM in vehicle, 25 mM OPLS, or 50 mM OPLS. Dots represent individual mice and bars are mean ± SD. Statistical significance was determined by Student’s unpaired t-test (one-tailed). *p<0.05, **p<0.01.

Plasmablast differentiation and selection to become LLPCs within peripheral lymphoid compartments involves help from T cells (43), thus reduction of T cell activation may provide a mechanism by which OPLS reduced the frequency of CD138^+^ plasma cells. Lymphocyte activation can be identified by multiple markers, and high expression of CD44 (CD44^++^) is considered a marker for activation of memory effector T cells (44). For RTX + rHuPH20, the frequency of CD44^++^ CD4^+^ T cells in the draining lymph node (DLN) decreased with OPLS co-administration compared to vehicle (**Figure 4A**; **Supplementary Figure 3**). Prolonged stimulation of T cells by antigen can lead to exhaustion and upregulation of immune checkpoints, like CTLA-4 and PD-1, to negatively regulate T-cell activation. A significant difference in the frequency of CD4^+^ T cells expressing classical immune checkpoint molecules CTLA-4 and PD-1 was observed between the vehicle group and 100 mM OPLS group (p=0.030) (**Figure 4B**). ADM co-formulated with OPLS indicated similar findings of significantly reduced frequency of CTLA-4^+^PD-1^+^ CD4^+^ T cells in the DLN compared to vehicle group for 25 mM OPLS (p=0.002) and 50 mM OPLS (p=0.009) (**Figure 4C**). In the effective dose range, between 25 and 100 mM, OPLS likely prevented prolonged T-cell activation by therapeutic proteins, leading to reduced ADA development, limited plasma cell differentiation, and less T-cell exhaustion.

### 3.6 OPLS induces regulatory T cell phenotypes *in vitro* and *in vivo*

**Figure 5:**
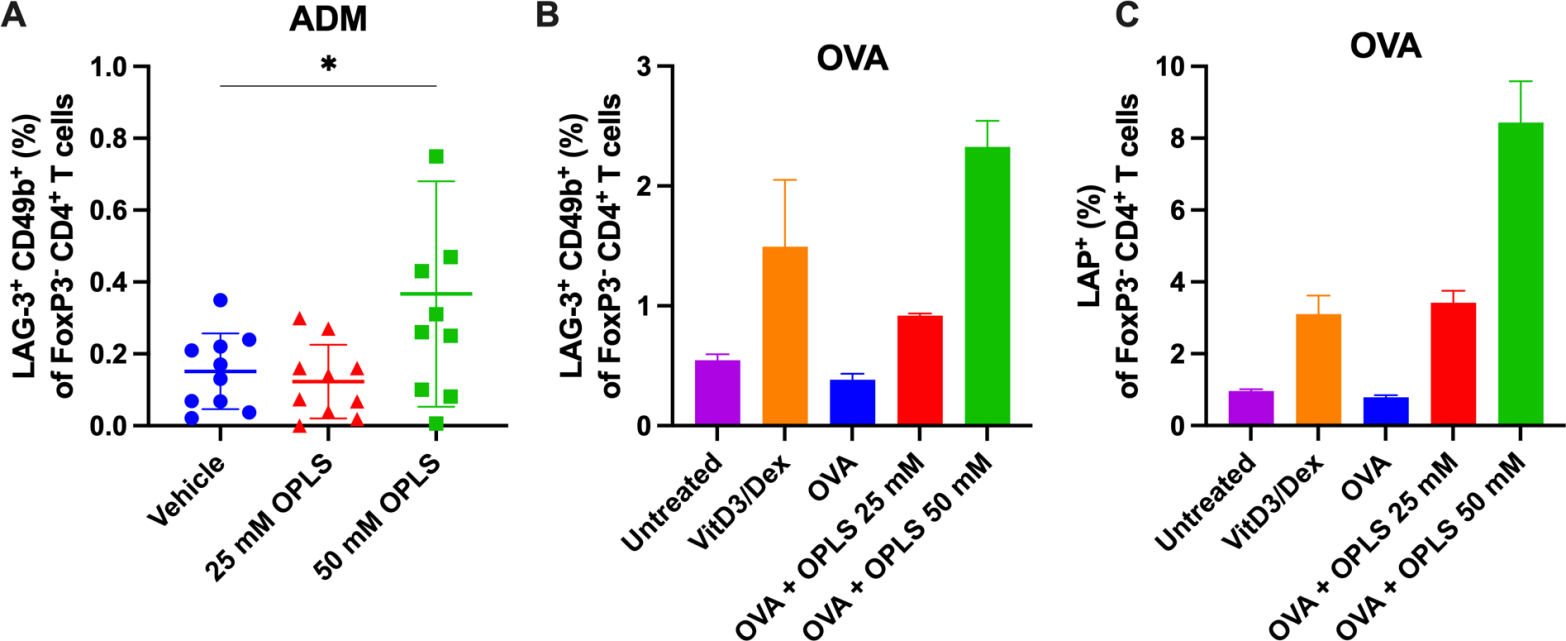
Co-administration with OPLS promotes differentiation of regulatory T cells *in vivo* and *in vitro*. In the ADM study (**Figure 1**), mouse splenocytes were collected at the terminal endpoint and analyzed for Tr1 phenotype. **Supplementary Figure 4** is the flow cytometry gating strategy. **(A)** Frequency (%) of LAG-3^+^CD49b^+^ FoxP3^-^CD4^+^ Tr1 cells in the spleen. Dots represent individual mice and bars are mean ± SD. Statistical significance was determined by Student’s unpaired t-test (one-tailed). *p<0.05. **(B, C)** Naïve immature SW mouse BMDCs were cultured for 24 h with media, VitD3/Dex, OVA (a model antigen), and OVA + 25 mM or 50 mM OPLS. Splenocytes from SW mice (n=2) subcutaneously immunized with OVA (2 μg/100 μL) once weekly were collected 4 days after the second dose. Isolated CD4^+^ T cells were co-cultured with treated BMDCs at a ratio of 1:5 BMDC, CD4^+^ T cell for 72 h. Frequency (%) **(B)** LAG-3^+^CD49^+^ Tr1 and **(C)** LAP^+^ of CD4^+^FoxP3^-^ T cells. Error bars are mean ± SD of triplicate wells.

**Figure 6:**
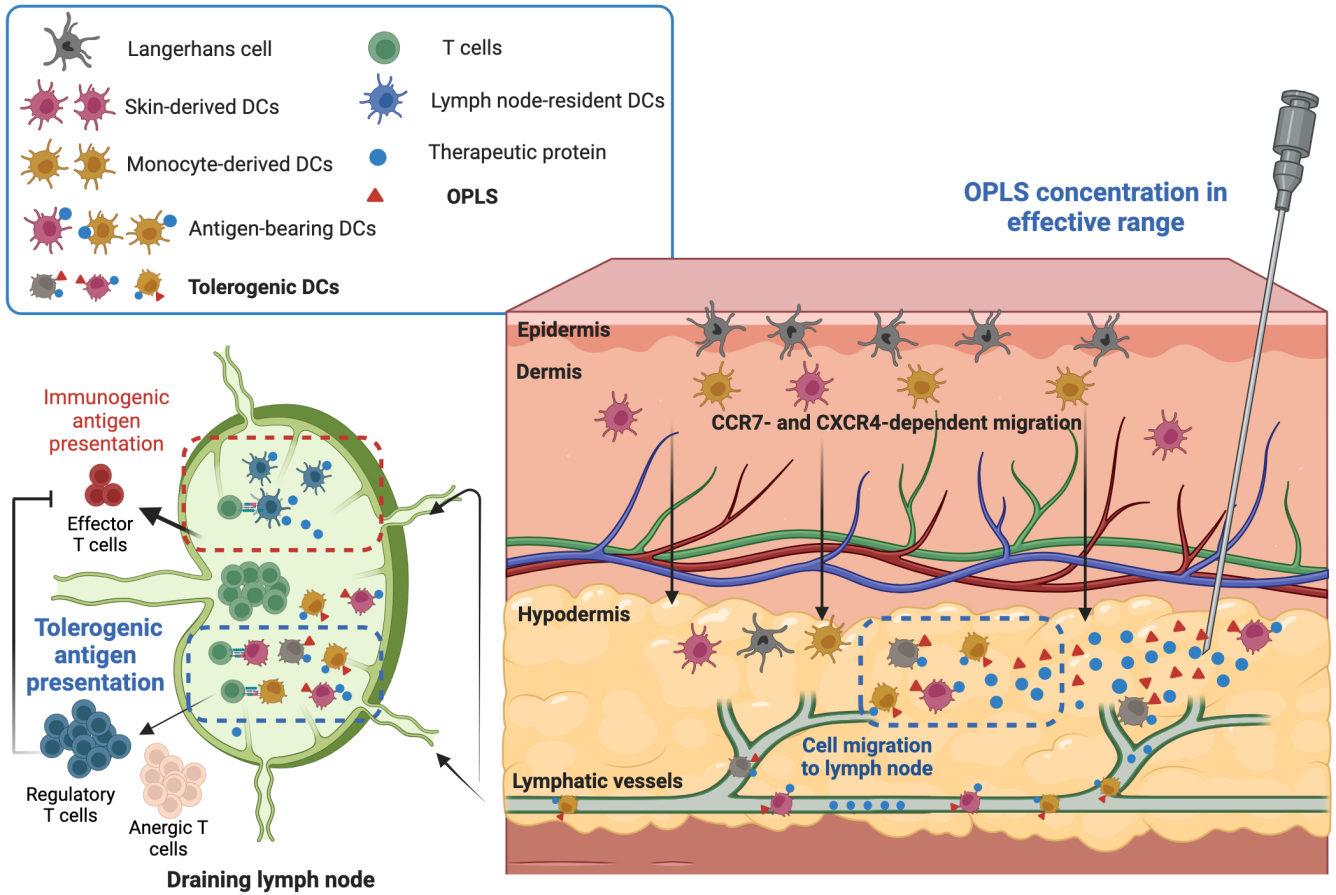
Proposed mechanism of OPLS immunogenicity reduction. In the effective dosage range (25-100 mM), OPLS signaling to local DCs during antigen uptake/processing induces a tolerogenic DC phenotype, leading to DC migration to the DLN. Antigen presentation by OPLS-primed DCs is more likely to induce tolerogenic or anergic T-cell responses than an immunogenic response. The reduction in ADA development by OPLS likely stems from less T cell help in humoral responses and/or restriction of T cell effector responses by regulatory T cells. This figure was created at Biorender.com.

OPLS may be able to induce immune regulation as it is structurally derived from the tolerogenic lipid PS (9). In the ADM study, the induction and/or recruitment of regulatory T cell (T_reg_) activity, specifically Tr1 cells, by OPLS was investigated. Tr1 cells in the spleen were gated based on co-expression of LAG-3 and CD49b which serves as a marker for Tr1 cells in both human and mouse subjects (**Supplementary Figure 4**) (45). Here, the group treated with ADM + 50 mM OPLS showed an induction of LAG-3^+^CD49b^+^ Tr1 cells (0.367%) within splenic FoxP3^-^CD4^+^ T cells as compared to ADM with vehicle (0.152%) (p=0.027) (**Figure 5A**). Next, in a preliminary *in vitro* experiment, the ability of OPLS-treated BMDCs to induce T_regs_ (Tr1 and LAP^+^) for a model protein antigen, ovalbumin (OVA), was investigated. SW mice were given SC injections of OVA (2 µg) once weekly for two weeks to enrich OVA-specific T cells. Then CD4^+^ T cells from mouse splenocytes were co-cultured with BMDCs that had been pretreated for 24 h with OVA (10 µg/mL) with and without OPLS (25, 50 mM). After 72 h, BMDCs treated with OVA and OPLS induced expression of the Tr1 phenotype (LAG-3^+^CD49b^+^) on viable CD4^+^FoxP3^-^ T cells compared to media or OVA alone (**Figure 5B**). The frequency of Tr1 cells increased from 0.38% in the OVA group to 0.92% for OVA + OPLS 25 mM and 2.3% for OVA + OPLS 50 mM. For comparison, the tolerogenic control treatment of vitamin D3 plus dexamethasone (VitD3/Dex) induced a frequency of 1.5% Tr1 cells (46) (**Figure 5B**). There was also an increase in viable CD4^+^FoxP3^-^ T cells expressing latency associated peptide (LAP) (47) from 0.79% for OVA alone to 3.4% and 8.4% for OVA + OPLS 25 or 50 mM, respectively (**Figure 5C**). Collectively, antigen processing by DCs in the presence of OPLS signaling induces a tolerogenic phenotype that could induce peripheral tolerance (**Figure 6**).

### 3.7 OPLS does not induce changes in immunocompetence in non-human primates and mice

**Figure 7:**
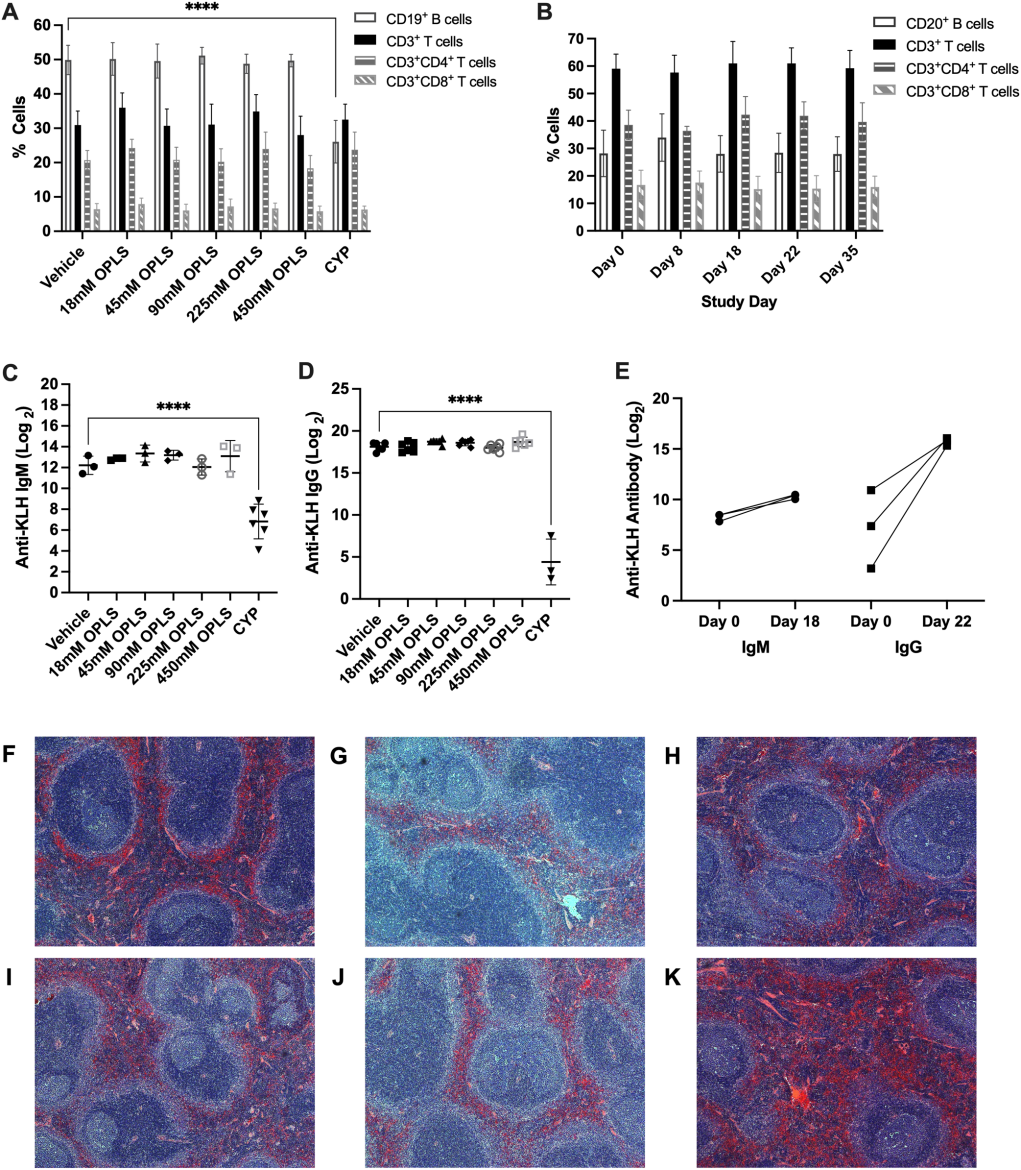
OPLS does not impact immunocompetence of NHP and mice. CD-1 mice received daily SC doses of vehicle (n=6), 20 mg/kg CYP (n=3), or OPLS (n=6/group) at 18-450 mM for 28 days. NHP (rhesus macaques) (n=3) received 21 daily SC doses of 54 mM OPLS. **(A, B)** Frequencies of lymphocyte populations—CD19^+^ B cells, CD3^+^ T cells, CD3^+^CD4^+^ T cells, and CD3^+^CD8^+^ T cells—in **(A)** spleens of mice on day 28 and **(B)** peripheral blood of NHP on day 0 (baseline), 8, 18, 22, and 35. **Supplementary Figure 5** is the gating strategy. **(C)** Anti-KLH IgM and **(D)** IgG titers (log_2_) in plasma of mice on day 28 following a single IV dose of KLH (2 mg) on day 15. **(E)** Anti-KLH IgM and IgG titers (log_2_) in plasma of NHP on day 18 and 22, respectively, following a single IM dose of KLH (10 mg) on day 8. Each dot represents an individual animal and all bars are mean ± SD. **(F-K)** Microscopic images of H&E-stained spleens from mice treated with **(F)** vehicle or **(G)** 18 mM, **(H)** 45 mM, **(I)** 90 mM, **(J)** 225 mM, and **(K)** 450 mM OPLS. Statistical significance was determined by one-way ANOVA with Dunnett’s multiple comparisons test. ****p<0.0001.

Two species toxicity studies were conducted in CD-1 mice and rhesus macaque NHPs to investigate toxicity of OPLS in multiple organ systems up to 583 mg/kg (450 mM) (**Supplementary Tables 1**, **2**). First, immunophenotyping of murine splenic lymphocytes and NHP peripheral blood evaluated the effect of OPLS on overall immunocompetence. No detectable changes were observed in blood cell counts of any OPLS-treated animals relative to the vehicle in mice (**Supplementary Table 3**) and the baseline in NHP (**Table 1**). Values fell within reference ranges for male rhesus macaques provided by TNPRC and for CD-1 mice in literature (48). In comparison, mice treated with the positive control, 20 mg/kg of cyclophosphamide monohydrate (CYP), had much lower levels of lymphocytes. Immunophenotyping revealed no changes in splenic lymphocyte subsets of OPLS-treated mice relative to the vehicle: CD19^+^ B cells, CD3^+^ T cells, CD3^+^CD4^+^ T cells, and CD3^+^CD8^+^ T cells (**Figure 7A**). Whereas the positive control group, CYP-treated mice, had a significant decrease in the percentage of B cells (p<0.0001). Similar results were observed in peripheral blood from OPLS-treated NHP; no temporal changes in lymphocyte subpopulations were observed relative to baseline (**Figure 7B**). Next, T-cell dependent antibody response (TDAR) assays were conducted in both species. Mice were immunized with 2 mg KLH by IV injection and NHP were immunized with 10 mg KLH by IM injection. Mean anti-KLH IgM and IgG titers in all murine OPLS treatment groups were similar to mean titer levels of the vehicle group (**Figures 7C, D**). However, the positive control CYP-treated group had significantly lower mean titer levels. OPLS-treated NHP also mounted a robust anti-KLH IgG response relative to baseline levels (**Figure 7E**). Macroscopic examinations of splenic morphology in mice revealed no gross deviations compared to vehicle even at the highest dose of OPLS (**Figures 7F-K**). Mild enlargement of the axillary and inguinal lymph nodes was observed in OPLS-treated NHP beginning at day 18 of treatment that was resolved within two weeks of treatment cessation. Results in both species suggest that OPLS did not impact the immunocompetence of animals up to 583 mg/kg (450 mM).

**Table 1:** Peripheral blood cell counts of rhesus macaques treated with 25 mg/kg (54 mM) OPLS.

Parameter	Mean ± SD		Reference Range
	Day 0	Day 22	
White blood cells (x 10^9^/L)	6.2 ± 2.0	8.2 ± 1.7	6.60 – 15.5
Lymphocytes (x 10^9^/L)	3.4 ± 0.9	3.0 ± 1.1	2.30 – 13.0
Monocytes (x 10^9^/L)	0.2 ± 0.0	0.3 ± 0.1	0.10 – 1.50
Neutrophils (x 10^9^/L)	2.3 ± 1.3	4.7 ± 1.7	1.40 – 7.30
Eosinophils (x 10^9^/L)	0.2 ± 0.1	0.2 ± 0.1	0.00 – 0.80
Basophils (x 10^9^/L)	0.02 ± 0.0	0.03 ± 0.02	0.00 – 0.80
Lymphocytes %	55.8 ± 7.1	37.1 ± 12.9	35.2 – 84.1
Monocytes %	4.2 ± 1.2	3.8 ± 1.1	1.90 – 9.90
Neutrophils %	35.2 ± 9.2	56.3 ± 15.3	20.6 – 46.9
Eosinophils %	4.4 ± 2.4	2.5 ± 1.4	0.00 – 5.30
Basophils %	0.4 ± 0.2	0.4 ± 0.2	0.00 – 0.60
Red blood cells (x 10^12^/L)	5.6 ± 0.4	5.3 ± 0.2	4.10 – 7.80
Hemoglobin (g/dL)	13.7 ± 1.0	13.1 ± 0.4	10.1 – 15.9
Hematocrit (%)	42.4 ± 3.3	39.4 ± 1.8	34.8 – 55.2
Mean corpuscular volume (fL)	75.3 ± 1.4	74.3 ± 1.5	63.7 – 86.9
Mean corpuscular hemoglobin (pg)	24.4 ± 0.2	24.7 ± 0.1	19.1 – 27.7
Mean corpuscular hemoglobin concentration (g/dL)	32.4 ± 0.4	33.2 ± 0.6	28.9 – 35.4
Red cell distribution width (%)	12.4 ± 0.2	12.8 ± 1.0	10.9 – 15.3
Platelets (x 10^9^/L)	426 ± 118	432 ± 70	193 – 676
Mean platelet volume (fL)	5.8 ± 0.6	8.2 ± 1.2	7.00 – 12.0

Reference ranges were provided by TNPRC.

The presence of PS-reactive antibodies in OPLS-treated mice and NHP was also assessed since these would increase the risk of anti-phospholipid syndrome (APS) (49). No PS-reactive antibodies (IgM or IgG) were detected in plasma from any OPLS-treated NHP (**Supplementary Table 4**). No mice in the highest OPLS dose group (450 mM) developed PS-reactive antibodies; however, one mouse in the 225 mM OPLS group tested positive transiently for PS-reactive IgM antibodies which resolved in the following week (**Supplementary Table 5**). The animal that tested positive did not show any other diagnostic features of APS (49), such as thrombotic events, thrombocytopenia, or prolongation of plasma clotting time (as assessed by activated partial thromboplastin time measurements), suggesting that the development of these antibodies might represent a transient, non-pathological event.

### 3.8 OPLS does not induce renal or hepatic toxicity in non-human primates and mice

**Figure 8:**
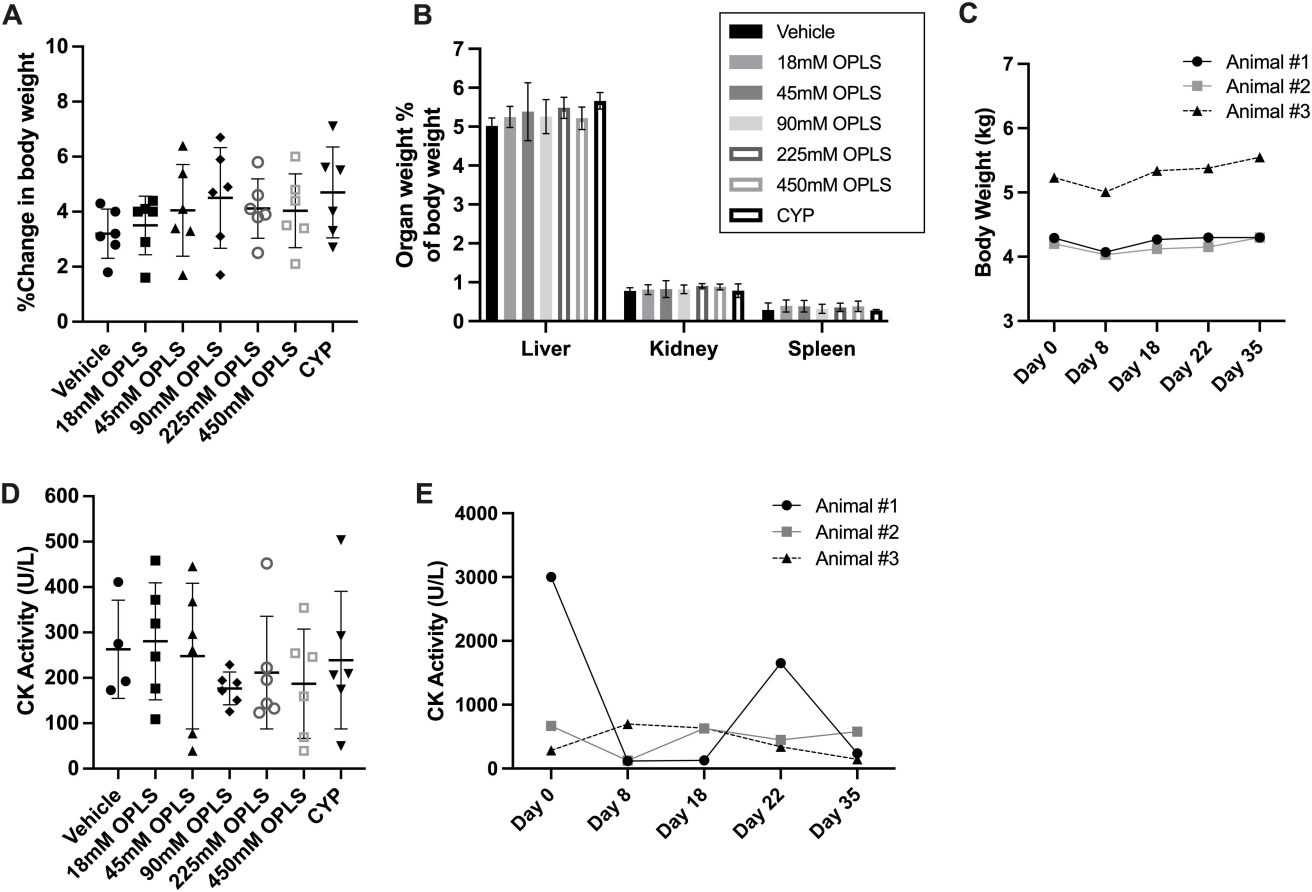
OPLS does not induce renal or hepatic toxicity in NHP and mice. **(A)** Change in body weight (%) from day 1 to day 28 and **(B)** organ weight (%) of body weight for the liver, spleen, and kidney at day 28 for CD-1 mice (n=6/group) administered daily SC doses of vehicle, 18-450 mM OPLS, or CYP (20 mg/kg). **(C)** Body weight (kg) of NHP (rhesus macaques) (n=3) administered daily SC doses of 54 mM (25 mg/kg) OPLS. **(D, E)** Creatinine kinase (CK) activity (U/L) in plasma of **(D)** CD-1 mice at day 28 and **(E)** NHP across study days.

NHP and mouse serum chemistry results are presented in **Table 2** and **Supplementary Table 6**, respectively, and indicate no evidence of OPLS-induced renal or hepatotoxicity. No differences in mean weights, gross and histological morphology of kidney, liver and spleen samples were observed between vehicle- and OPLS-treated animals (**Figures 8A–C**; **Supplementary Figure 6**). No physical signs of injection site toxicity were observed in any of the OPLS-treated mice or NHP. Histological examination of the injection site tissue from mice indicated no notable morphological damage (**Supplementary Figure 6**). Mean plasma creatinine kinase (CK) concentrations in all OPLS-treated mice were comparable to the vehicle-treated group (**Figure 8D**). Plasma CK concentrations fluctuated in one NHP during the course of treatment but declined to baseline levels within two weeks of the last dose (**Figure 8E**).

**Table 2:** Serum blood chemistry of rhesus macaques treated with 25 mg/kg (54 mM) OPLS.

Parameter	Mean ± SD		Reference Range
	Day 0	Day 22	
Albumin (g/dL)	4.2 ± 0.26	4.3 ± 0.14	3.00– 5.9
Globulin (g/dL)	1.97 ± 0.15	2.4 ± 0.0	1.9 – 3.9
Albumin/Globulin Ratio	2.17 ± 0.15	1.8 ± 0.0	0.5 – 3.5
Total Protein (g/dL)	6.17 ± 0.35	6.7 ± 0.14	5.9 – 7.8
Alkaline phosphatase (U/L)	517 ± 113	376 ± 18.4	55 – 649
Alanine aminotransferase (U/L)	34.0 ± 13.2	45.0 ± 2.83	20 – 126
Aspartate aminotransferase (U/L)	72.7 ± 68.3	44.0 ± 5.66	25 – 120
Lactate dehydrogenase (U/L)	852 ± 730	464 ± 31.8	129 – 644
Total bilirubin (mg/dL)	0.13 ± 0.06	0.09 ± 0.02	0.10 – 0.70
Blood urea nitrogen (mg/dL)	15.3 ± 2.52	15.5 ± 2.12	13 – 27
Creatinine (mg/dL)	0.63 ± 0.11	0.69 ± 0.11	0.40 – 1.40
Blood urea nitrogen**/**Creatinine ratio	25.3 ± 8.95	22.7 ± 0.42	11.0 – 60.0
Calcium (mg/dL)	9.37 ± 0.50	10.5 ± 0.14	9.4 – 12.2
Phosphorus (mg/dL)	6.13 ± 0.45	4.90 ± 0.14	3.4 – 7.5
Glucose (mg/dL)	77.3 ± 16.8	79.5 ± 17.7	48 – 119
Sodium (mmol/L)	148 ± 2.65	149 ± 2.83	144 – 160
Potassium (mmol/L)	4.17 ± 0.72	4.05 ± 0.07	3.3 – 6.4
Chloride (mmol/L)	110 ± 1.0	110 ± 2.83	106 – 117
Cholesterol (mg/dL)	161 ± 58.8	176 ± 35.4	106 – 241

Reference ranges were provided by TNPRC.

## 4 Discussion

Although the SC route of administration has many desirable advantages such as reduced time and cost, it introduces unique immunogenicity challenges for therapeutic proteins. Here, we demonstrated that OPLS can prevent or reduce ADA formation against multiple protein antigens in animal models, and it was identified that the reduction in titers stems from a reduction in plasma cell differentiation. Furthermore, OPLS was shown to prevent the overactivation of T cells compared to the vehicle group in which prolonged exposure to high concentrations of antigen led to T-cell activation and exhaustion with expression of checkpoint molecules CTLA-4 and PD-1. Results for all mAbs investigated suggest a reduction in relative immunogenic potential when administered subcutaneously in a formulation containing 25 to 100 mM OPLS. rHuPH20 was not strongly immunogenic in mice, likely due to limited absorption into systemic circulation and very short half-life (50, 51), but OPLS co-administration did reduce the relative immunogenicity of rHuPH20 based on lower titer development (**Figures 2D, F**). Co-formulation with OPLS for SC delivery could reduce the risk of immunogenic response toward not only the protein therapeutic but also the dispersion enhancer, rHuPH20, when it is used in the formulation.

In early proof-of-concept studies performed in Hemophilia A mice, recombinant human FVIII was formulated in buffer containing low concentrations of OPLS (i.e., 10 mM). OPLS is the key structural feature of phosphatidylserine (PS) that mediates membrane binding of FVIII to platelets (52). Thus, OPLS can complex to FVIII (K_d_ = 8 μM) and improve its stability (19). Furthermore, relative immunogenicity studies with FVIII demonstrated that 10 mM OPLS was sufficient to significantly reduce anti-FVIII antibody responses (18–20). *In vitro* studies have also demonstrated the induction of tolerogenic properties on DCs treated with 10 to 50 mM OPLS (21). For mAbs, a higher dose of OPLS, for example up to 100 mM, may be needed to see an effect since they are expected to have low binding or complexation with OPLS. Also, without complexation, co-administration is important to ensure simultaneous signaling of local DCs by OPLS and protein antigen to induce an antigen-specific tolerogenic phenotype. Furthermore, as compared to IV, the SC route of administration is better suited for OPLS immunomodulatory activity where protein movement is restricted in the extracellular matrix composed of collagen and hyaluronic acid (53). As shown in the proposed mechanism schematic in **Figure 6**, tissue-resident DCs primed with OPLS in the presence of protein antigen would then migrate to the draining lymph nodes where their T-cell activation pathway skews toward tolerogenic or anergic compared to immunogenic.

For TTZ and RTX formulated with 25 and 100 mM OPLS, respectively, there was a reduction in ADA concentration in the plasma which correlated with a reduction in the frequency of CD138^+^ plasma cells in the bone marrow compared to the vehicle groups. Plasma cells in the bone marrow represent a long-lived phenotype that continuously produces antibodies without requiring antigen stimulation. LLPCs are thought to arise from plasmablasts generated in germinal center (GC) reactions within secondary lymphoid organs (54). The current model suggests that plasmablasts with very high affinity are selected for this fate thus a strong BCR signal would be selected by T cells for differentiation. Plasmablasts that migrate through the blood to the bone marrow differentiate into LLPCs when they receive survival signals in a niche created by cells such as megakaryocytes, eosinophils, and monocytes (55). The ability of OPLS to reduce the magnitude of plasma cell differentiation seen for RTX and TTZ when formulated in vehicle is very promising. Plasma cells are particularly difficult to deplete due to their lack of common B-cell markers (e.g., CD20) and their location in the bone marrow survival niches (54, 56). Thus, plasma cells will continue to be a source of ADA following B-cell depletion. As a CD20 targeting agent, we do not expect the mechanism of action of RTX to have contributed to the reduced plasma cell frequency in OPLS-treated groups (57).

In the treatment group with the lowest plasma cell frequency (i.e. 100 mM OPLS), there were also significantly less CTLA-4- and PD-1-expressing CD4^+^ T cells in the draining lymph node (**Figure 4**). CTLA-4 and PD-1 act in non-overlapping mechanisms to inhibit T-cell activation and regulate immune homeostasis (58–60). These markers can be expressed by T_regs_; however, due to the trend in ADA development, it is unlikely the CTLA-4^+^ and PD-1^+^ CD4^+^ T cells that were highest in the vehicle groups represented T_reg_ subsets. Most likely, these are populations of exhausted T cells that were induced to negatively regulate T-cell activation from prolonged TCR stimulation by weekly high dose RTX (0.5 mg/mouse). In the ADM study, results also suggested reduced ADA development correlated with reduction in T cell exhaustion. Since most LLPCs derive from the GC reaction (55), local immune response can be further investigated by staining for intracellular proliferative lymphocyte markers indicative of germinal centers. Less T-cell help in GCs should result in reduced differentiation of plasmablasts into antibody-secreting plasma cells (43). ADA responses may also be suppressed by T_regs_ (61). The ability of OPLS to induce T_regs_ *in vivo* was not previously known, although *in vitro* studies have shown OPLS induces phenotypic properties on DCs that are likely to skew T-cell differentiation toward T_regs_ upon antigen presentation. Expression of MHC II is maintained but CD40 is reduced, and cytokine responses skew toward tolerogenic (increased IL-10, TGF-β) instead of proinflammatory (reduced IL-12p70 and TNF-α) (21). Here, T_reg_ induction was preliminarily probed *in vitro* in a DC:T-cell co-culture experiment in which an expansion of Tr1 cells (LAG-3^+^CD49b^+^) and LAP^+^ T_regs_ was observed upon treatment with OVA formulated with 25 or 50 mM OPLS (**Figure 5**). Furthermore, in the ADM relative immunogenicity study, a 2-fold induction of Tr1 cells was detected in mice co-administered ADM and 50 mM OPLS compared to ADM in vehicle.

Multiple species toxicity studies in mice and NHP proved OPLS to be well tolerated and that it does not alter the state of general immune competence. Daily doses of 25 mg/kg (54 mM) OPLS did not significantly alter normal frequencies of lymphocyte subsets in peripheral blood of NHP, and in CD-1 mice, OPLS doses up to 583 mg/kg (450 mM) did not induce general immune suppression. Both mice and NHP were able to mount robust T-cell mediated antibody responses against an unrelated antigen (KLH) administered at a distal site. No evidence of OPLS-induced renal or hepatotoxicity was detected as indicated by healthy macro- and microscopic morphology of these organs and normal concentrations of kidney and liver function markers in serum of mice and NHP. Furthermore, the potential for OPLS to cause damage to the injection site was investigated since hyperosmolar solutions can cause injection site tissue damage (62). No changes in injection site morphology were observed in mice and plasma concentrations of creatinine kinase, indicative of muscle tissue damage, were not significantly elevated in animals across the study period. Based on results of these studies, the proposed no observed adverse effect level (NOAEL) of OPLS in mice and primates is 583 mg/kg and 25 mg/kg, respectively. Assuming a 1 mL injection of 100 mM OPLS in a 60 kg human subject, the anticipated clinical dose of OPLS may be 0.31 mg/kg. With the application of allometric scaling and safety factor recommended by the FDA (63), the values from mice and primates translate to predicted safe starting doses in humans of 4.7 mg/kg and 0.8 mg/kg, respectively.

Future work on sequential administration of OPLS and protein will be tested to determine optimal timing and distance between injections. A sequential dosing regimen would simplify the use of OPLS without the requirement of a new therapeutic protein formulation, and the use of a microneedle patch or other dosage forms to locally deliver OPLS prior to protein injection may be possible. Overall, OPLS co-administration can reduce the relative immunogenicity of therapeutic proteins in a manner that is generally safe and preserves general immune system activity.

## Data Availability

The original contributions presented in this study are included in the article or **Supplementary Material**. Further inquiries can be directed to the corresponding author.
