# Supplementary material for: Immune regulatory adjuvant approach to mitigate subcutaneous immunogenicity of monoclonal antibodies
Source: Front Immunol. 2024 Dec 10;15:1496169. doi: 10.3389/fimmu.2024.1496169 (PMC11666448; doi:10.3389/fimmu.2024.1496169)
Supplement: Supplementary file 1 [file DataSheet1.pdf]

Immune Regulatory Adjuvant Approach to Mitigate Subcutaneous Immunogenicity of Monoclonal Antibodies

Supplementary Material

1 Supplementary Methods

1.1 Anti-KLH Antibody ELISA

Anti-KLH IgM and IgG antibodies were detected by ELISA. Briefly, 96-well microtiter plates were coated with KLH and following a blocking step, two-fold serial dilutions of samples (beginning at 1:100 for murine IgM samples and 1:1000 for murine IgG and NHP samples) were applied. Dilutions (1:100 or 1:1000 fold) of naïve, pooled plasma from the respective species (8 wells/plate) were included on each plate as negative controls. Bound anti-KLH antibodies were detected using anti-mouse or anti-rhesus IgM and IgG detection antibodies. The negative cutoff for each plate was defined as the mean plus 2 standard deviations of the absorbance values of the negative controls on the given plate and was used to estimate endpoint titers (reported as $\log_2$).

1.2 Anti-rHuPH20 Antibody ELISA

Nunc™ Maxisorp™ 96-well plates were coated with 5 µg/mL rHuPH20 (Creative Enzymes, Shirley, NY) in 50 µL/well PBS and incubated at 37°C for 2 h. The plate was washed 4 times with wash buffer (0.05% Polysorbate 20 in PBS) on an automated plate washer. The plate was blocked with 300 µL/well of diluent (0.05% Polysorbate 20 and 1% BSA in PBS) for 1 h at 37°C. Plasma samples were thawed on ice and mixed well before diluting 1:10 in diluent followed by 3-fold serial dilution until 1:21780. Naïve control plasma samples (n=4) were diluted 1:1000 in diluent and the positive control, immunized plasma sample was diluted 1:100 in diluent. After washing, samples and standards were plated 100 µL/well in duplicate followed by incubation for 2 h at 37°C. The detection antibody, goat anti-mouse IgG Fc-HRP (Southern Biotech, Birmingham, AL), was diluted 1:5000 in diluent. The plate was washed and 100 µL detection antibody was added to all wells for 1 h at 37°C. After washing, 100 µL/well of TMB substrate was added. Color development proceeded for 35 min at room temperature (RT) before adding 100 µL/well 2N H_2SO_4. Absorbance readings at 450 nm were background corrected by subtracting the absorbance of diluent samples. Anti-rHuPH20 IgG titers were calculated by the Frey Method as the reciprocal dilution where the absorbance meets the cutoff (cutoff = $\bar{X} + SD*f$). $\bar{X}$ and SD are the average and standard deviation, respectively, of the absorbance values for naïve plasma. f is a t-statistic based on the number of negative control samples (n=4) and the confidence level (95%). For this data set, $f = 2.631$. The curve for each sample was fitted with a power curve ($y = A*x^{-b}$) in GraphPad Prism and the reciprocal dilution value "x" was interpolated at y = cutoff.

1.3 Anti-RTX, Anti-TTZ, and Anti-ADM Antibody ELISAs

Nunc™ Maxisorp™ 96-well plates were coated with 2 µg/mL RTX, 2 µg/mL TTZ, or 1 µg/mL ADM in 100 µL/well PBS at 4°C overnight. The plate was washed 5 times with wash buffer (0.05% Polysorbate 20 in PBS) on an automated plate washer. 300 µL/well of diluent (0.05% Polysorbate 20

and 5% BSA in PBS) was added and incubated at RT for 1-1.5 h. Plasma samples were thawed on ice and mixed well before diluting 1:100 or 1:200 in diluent. A standard curve was prepared with anti-RTX (clone 6C1), anti-TTZ antibody (clone 11C4), or anti-ADM (clone 3C2) antibody in diluent from 0.488 to 2000 ng/mL. After washing, samples and standards were plated at 100 µL/well in triplicate and incubated at RT for 1 h. The detection antibody, goat anti-mouse IgG Fc-HRP, was diluted 1:10,000 in diluent. After washing, 100 µL detection antibody was added to all wells and incubated at RT for 1 h. The plate was washed 10 times and 100 µL/well of TMB substrate was added. Color development proceeded for 7 min at RT before adding 100 µL/well 2N H_2SO_4. Absorbance readings at 450 nm were background corrected by subtracting the absorbance of diluent samples. The concentration (ng/mL) of anti-RTX, anti-TTZ, or anti-ADM IgG antibody was interpolated from the respective standard curve using the 4-parameter logistic (4-PL) function in GraphPad Prism.

1.4 Anti-Lipid Antibody ELISA

The presence of PS-reactive antibodies in plasma of CD-1 mice and rhesus macaques was detected using a lipid-adapted ELISA. Microtiter plates were coated with PS by adding 50 µL of a 20 µg/mL ethanolic solution of PS to each well and allowing the solvent to evaporate overnight. Plates were then saturated with a 10% solution of FBS in PBS (FBS-PBS). Samples and controls diluted 1:50 with FBS-PBS were then applied and allowed to incubate at RT for 1 h. An anti-PS IgG antibody spiked into naïve mouse plasma and diluted similarly was run on each plate (for IgG antibody analyses) as a positive control. Bound antibodies were detected using anti-mouse or anti-rhesus IgM and IgG detection antibodies. Plates were washed between steps three times with PBS. Samples with absorbance values greater than 3 times the average absorbance of the negative controls were considered positive for PS-reactive antibodies.

2 Supplementary Figures and Tables

2.1 Supplementary Figures

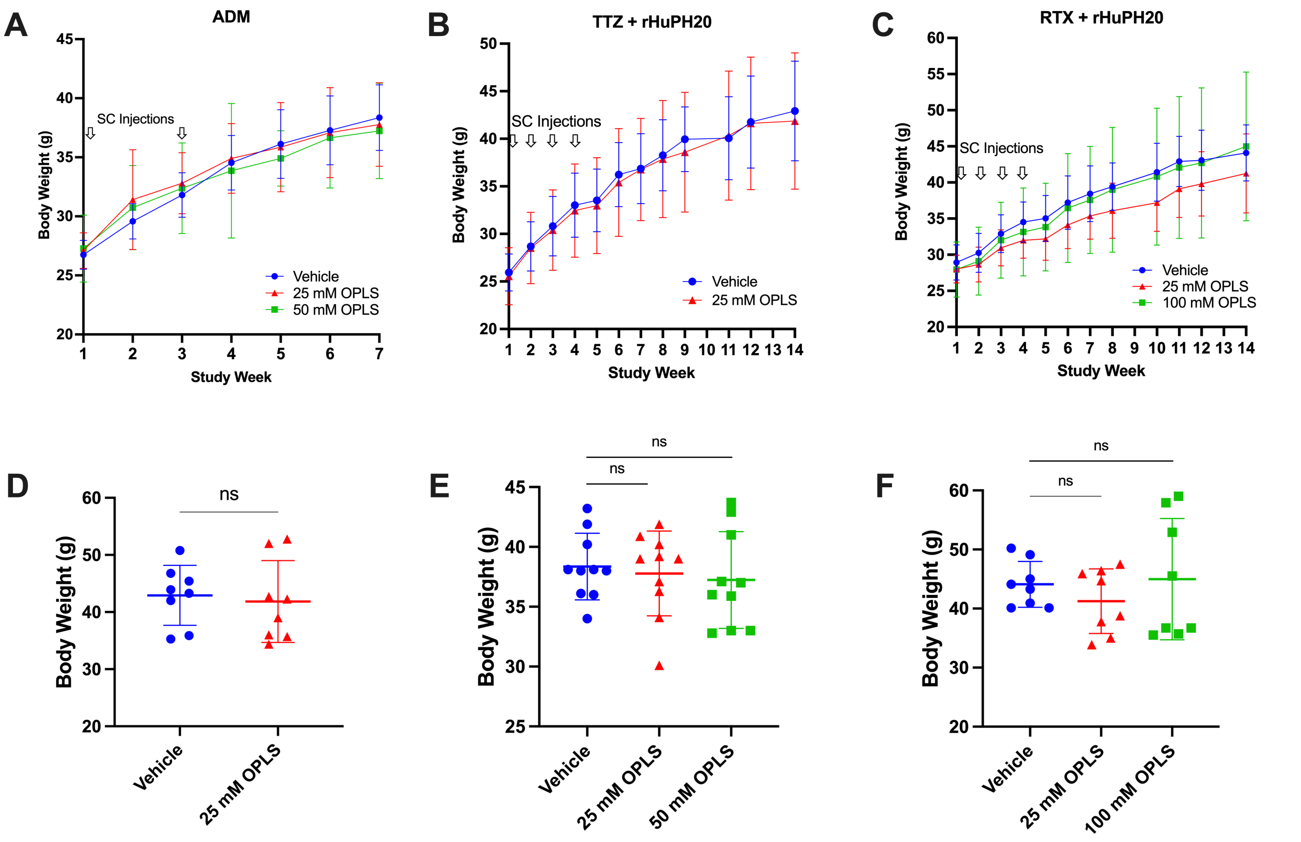

Supplementary Figure 1. OPLS co-administration does not impact mouse body weight. **(A)** Mouse body weight across study weeks for groups administered ADM formulated in vehicle or 25 mM and 50 mM OPLS (n=10/group). **(B)** Mouse body weight across study weeks for groups administered TTZ and rHuPH20 formulated in vehicle or 25 mM OPLS (n=8/group). **(C)** Mouse body weight across study weeks for groups administered RTX and rHuPH20 formulated in vehicle, 25 mM or 100 mM OPLS (n=8/group). Mean body weights at the terminal were compared between groups administered **(D)** TTZ, **(E)** ADM, and **(F)** RTX with and without OPLS, and statistical significance was determined by student's unpaired t-test (one-tailed). *ns* not significant ($p > 0.05$). All bars are mean ± SD.

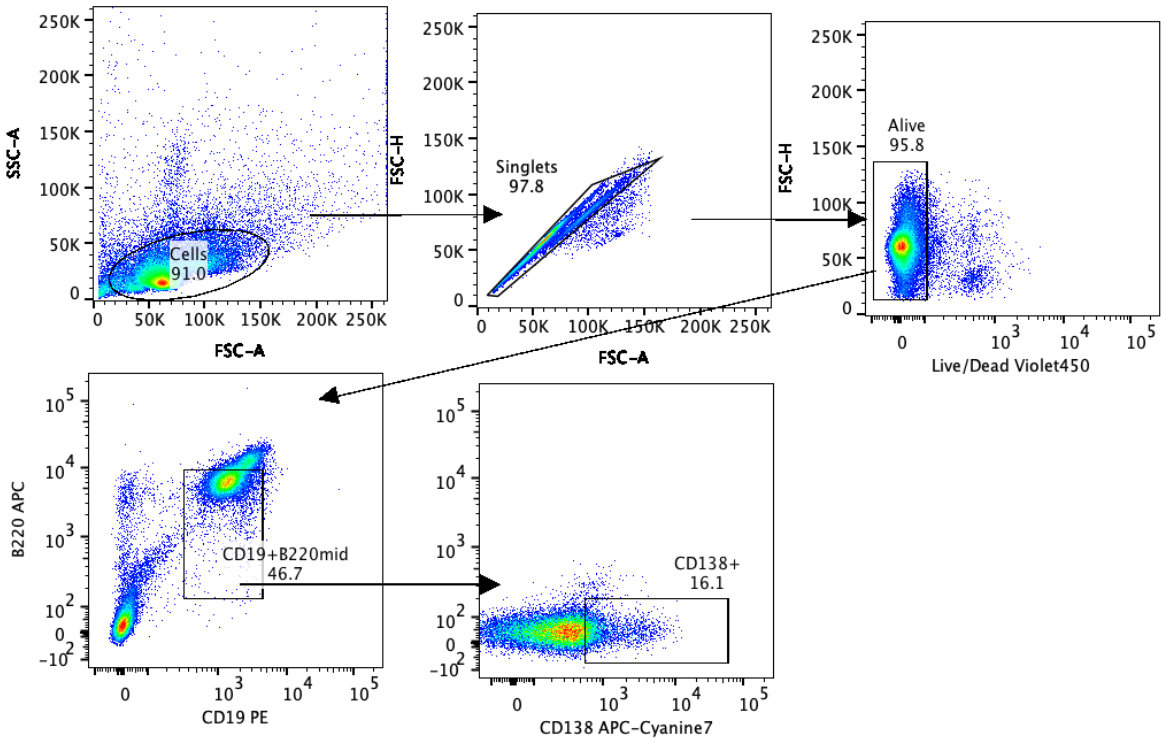

Supplementary Figure 2. Representative flow cytometry gating strategy for CD138+ plasma cells in the bone marrow of mice. CD138+ events were gated on an FMO control.

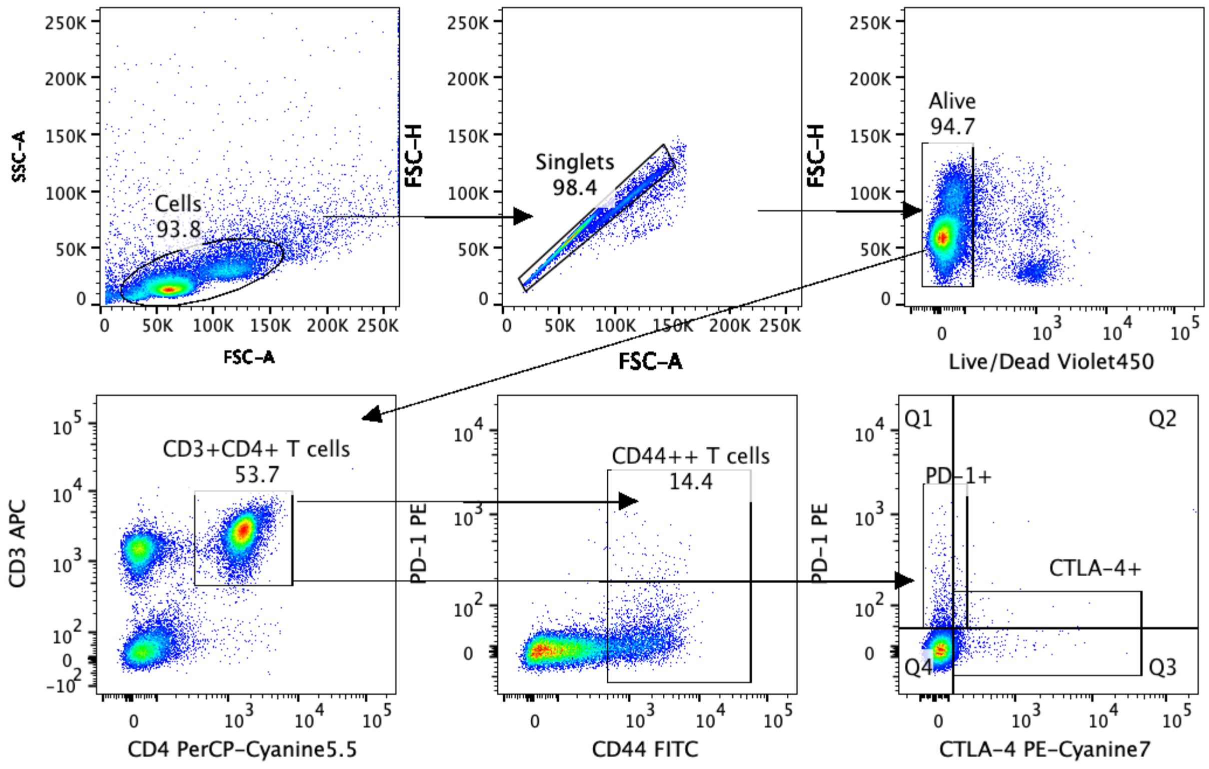

Supplementary Figure 3. Representative flow cytometry gating strategy for activated/exhausted CD4$^+$ T cells in the draining lymph node of mice. CD44$^+$, PD-1$^+$, and CTLA-4$^+$ events were gated on the associated FMO controls.

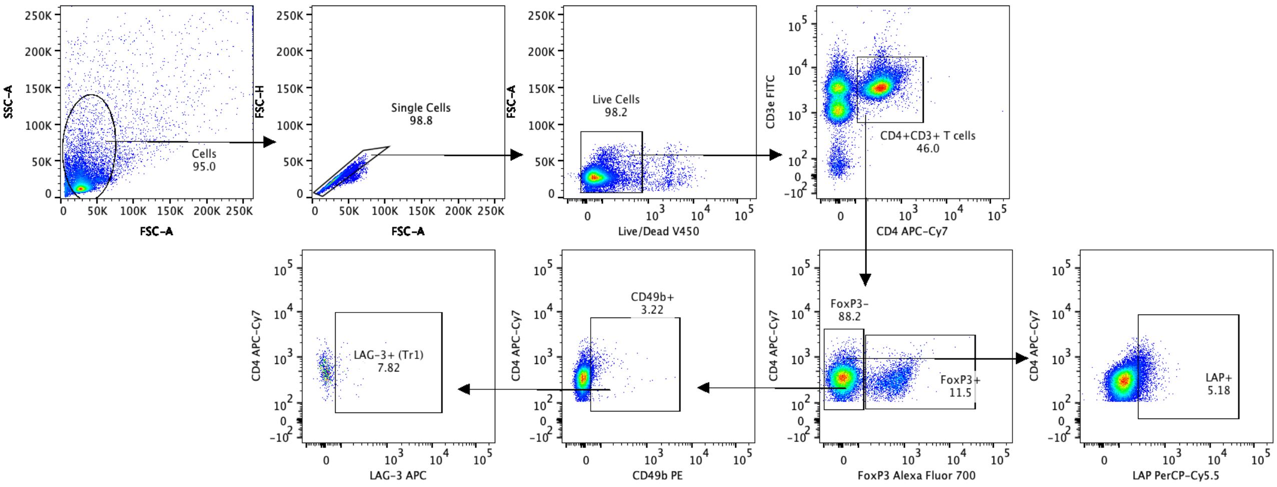

Supplementary Figure 4. Representative flow cytometry gating strategy for regulatory T cells (LAG-3^{+}CD49b^{+} Tr1 and LAP^{+}). LAP^{+}, CD49b^{+}, and LAG-3^{+} events were gated on isotype controls.

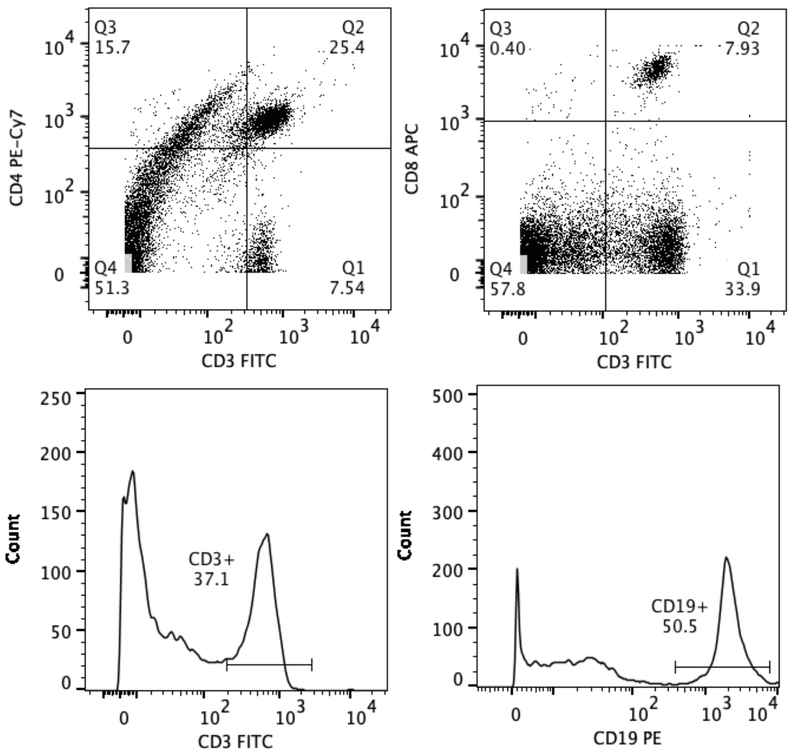

Supplementary Figure 5. Flow cytometry gating strategy for immunophenotyping in OPLS two species toxicity studies.

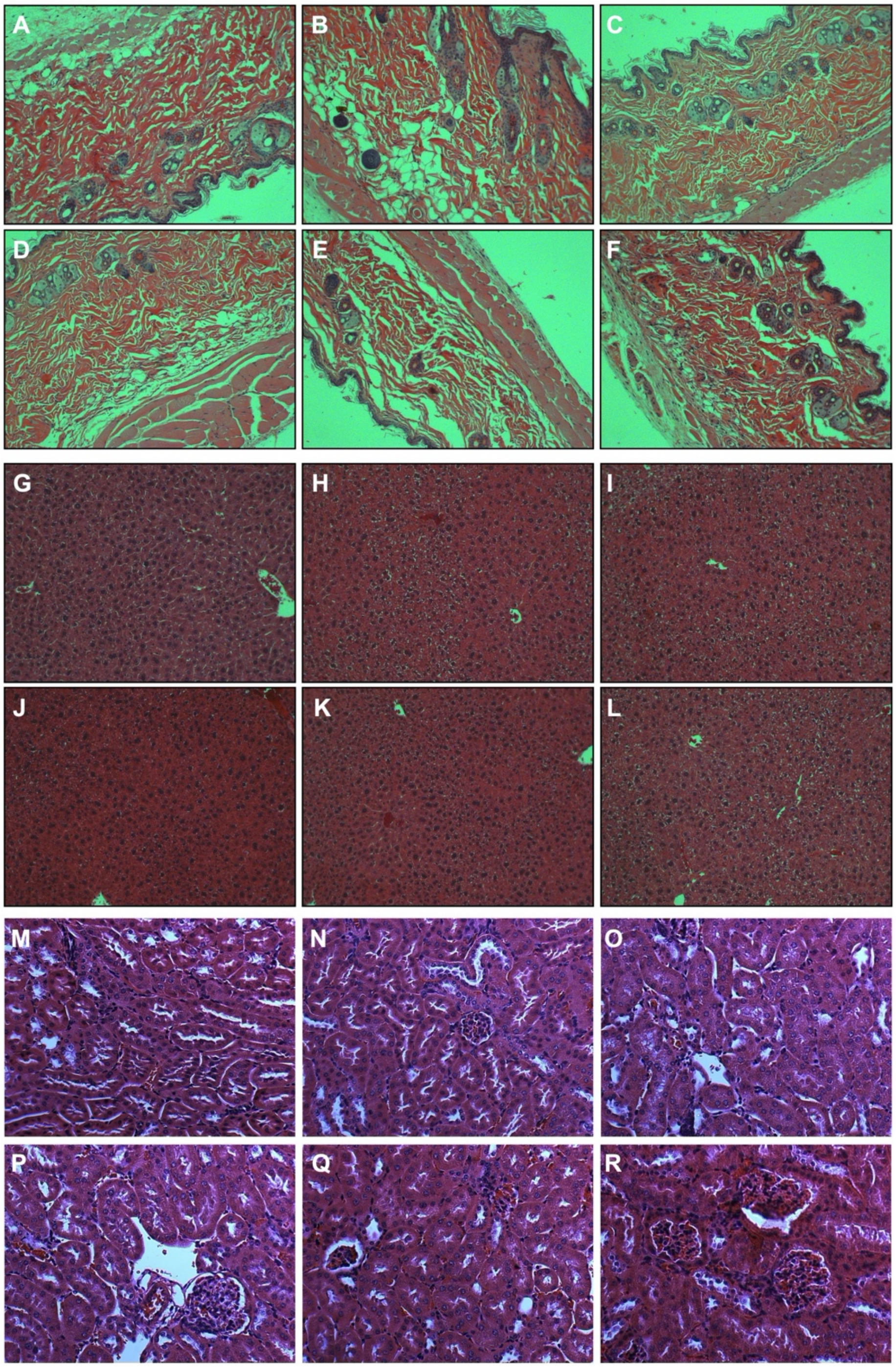

Supplementary Figure 6. Histology of liver, kidney, and skin injection site in CD-1 mice on day 28 after daily SC doses of OPLS. (**A-F**) Microscopic images of H&E-stained injection site tissue samples from representative mice administered (**A**) vehicle or (**B**) 18 mM, (**C**) 45 mM, (**D**) 90 mM, (**E**) 225 mM, and (**F**) 450 mM OPLS. (**G-L**) Microscopic images of H&E-stained liver tissue samples from representative mice administered (**G**) vehicle or (**H**) 18 mM, (**I**) 45 mM, (**J**) 90 mM, (**K**) 225 mM, and (**L**) 450 mM OPLS. (**M-R**) Microscopic images of H&E-stained kidney tissue samples from representative mice administered (**M**) vehicle or (**N**) 18 mM, (**O**) 45 mM, (**P**) 90 mM, (**Q**) 225 mM, and (**R**) 450 mM OPLS.

2.2 Supplementary Tables

Supplementary Table 1. Toxicity endpoints for mouse and non-human primate studies.

Organ/system	Toxicity endpoints	Study species
Immune system	TDAR study: anti-KLH IgM and IgG titers	Mice, NHP
	Peripheral blood cell counts	Mice, NHP
	Lymphocyte immunophenotyping	Mice, NHP
	Lymphoid organ structure: macro- and microscopic evaluation	Mice
	Anti-lipid antibodies	Mice, NHP
Liver	Liver structure: macro- and microscopic evaluation	Mice
	Liver function	Mice, NHP
Kidney	Kidney structure: macro- and microscopic evaluation	Mice
	Kidney function	Mice, NHP
Injection-site reactions	Injection site: physical and microscopic evaluation	Mice, NHP (physical only)
	Plasma CK	Mice, NHP

Supplementary Table 2. Toxicity study treatment groups.

	OPLS (mg/kg/day)	OPLS (mM)	CYP (mg/kg/day)	No. of animals
Mice	0 (vehicle)	0 (vehicle)	-	6
	24	18	-	6
	55	45	-	6
	114	90	-	6
	276	225	-	6
	583	450	-	6
	-	-	20	3
NHP	25	54	-	3

Supplementary Table 3. Peripheral blood cell counts of CD-1 mice treated with OPLS. Values are presented as mean (SD). Reference ranges were sourced from literature (reference provided in main text).

Parameter	Treatment Group							Reference Range
	Vehicle	OPLS					CYP	
		18 mM	45 mM	90 mM	225 mM	450 mM		
White blood cells (x 10^9/L)	20.3 (7.5)	18.7 (4.4)	13.5 (7.7)	15.6 (4.6)	12.9 (0.3)	12.1 (4.7)	8.32 (2.6)	7.06 - 17.29
Lymphocytes (x 10^9/L)	16.7 (6.9)	13.6 (2.8)	8.6 (4.9)	12.3 (3.4)	9.97 (0.8)	9.37 (3.6)	4.37 (1.5)	5.01 - 11.6
Monocytes (x 10^9/L)	0.53 (0.2)	0.67 (0.4)	0.50 (0.3)	0.47 (0.3)	0.40 (0.2)	0.43 (0.2)	0.48 (0.1)	0.09 - 0.63
Granulocytes (x 10^9/L)	3.10 (0.4)	4.37 (2.3)	4.43 (2.8)	2.83 (1.0)	2.53 (0.8)	2.27 (1.0)	3.47 (1.1)	0.98 - 6.90
Lymphocytes %	81.1 (4.0)	74.0 (10.0)	62.7 (6.8)	79.1 (2.4)	77.1 (6.7)	77.7 (2.7)	52.3 (4.8)	37.5 - 85.0
Monocytes %	2.87 (0.3)	3.57 (1.5)	4.07 (0.5)	2.93 (0.6)	3.20 (1.0)	3.53 (0.3)	5.97 (1.7)	2.84 - 13.09
Granulocytes %	16.0 (3.7)	22.5 (8.5)	33.2 (6.5)	35.6 (30)	19.7 (5.7)	18.8 (2.4)	43.4 (6.1)	8.74 - 55.7
Red blood cells (x 10^{12}/L)	10.2 (0.2)	10.3 (0.3)	9.3 (2.4)	8.77 (1.2)	10.2 (0.4)	8.89 (2.1)	8.18 (0.5)	8.49 - 10.43
Hemoglobin (g/L)	164 (4.4)	152 (2.1)	136 (33)	137 (22)	155 (4.9)	134 (31)	116 (9.0)	13.8 - 16.8
Hematocrit (%)	44.0 (5.1)	39.3 (2.9)	37.8 (12)	40.3 (8.5)	38.7 (1.8)	33.3 (8.0)	34.6 (2.2)	39.3 - 47.0
Mean corpuscular volume (fL)	43.0 (4.1)	38.2 (2.0)	40.2 (2.5)	45.5 (4.3)	37.9 (0.6)	37.5 (0.1)	42.4 (2.1)	42.3 - 48.0
Mean corpuscular hemoglobin (pg)	16.0 (0.3)	14.8 (0.2)	14.6 (0.6)	15.5 (0.6)	15.1 (0.1)	15.0 (0.2)	14.2 (0.7)	14.7 - 17.5
Mean corpuscular hemoglobin concentration (g/L)	375 (35.1)	388 (24)	366 (37)	344 (27)	399 (6.7)	402 (6.0)	336 (25)	341 - 367
Red cell distribution width (%)	11.0 (0.1)	12.7 (0.5)	11.3 (1.0)	11.2 (0.5)	11.2 (0.8)	11.8 (1.1)	14.4 (0.6)	15.1 - 18.9
Platelets (x 10^9/L)	667 (47.8)	578 (159)	503 (283)	565 (54)	499 (28.6)	631 (288)	713 (186)	784 - 1812
Mean platelet volume (fL)	5.90 (0.1)	5.57 (0.1)	6.07 (0.4)	5.87 (0.2)	5.87 (0.2)	5.63 (0.1)	5.80 (0.2)	4.3 - 5.8

Supplementary Table 4. Anti-phosphatidylserine (PS) antibody results of rhesus macaques treated with 25 mg/kg (54 mM) OPLS.

Anti-PS IgG (absorbance at 405 nM)						
**Primate**	**Day 0**	**Day 8**	**Day 18**	**Day 22**	**Day 35**	**Negative Control**
1	0.507	0.541	0.628	0.686	0.655	0.491
2	0.516	0.515	0.698	0.763	0.478	
3	0.381	0.347	0.431	0.493	0.659	
**Absorbance / Cutoff[a]**						
**Primate**	**Day 0**	**Day 8**	**Day 18**	**Day 22**	**Day 35**	
1	0.344	0.367	0.426	0.466	0.445	
2	0.350	0.350	0.474	0.518	0.325	
3	0.259	0.235	0.293	0.334	0.447	
**Anti-PS IgM (absorbance at 405 nM)**						
**Primate**	**Day 0**	**Day 8**	**Day 18**	**Day 22**	**Day 35**	**Negative Control**
1	1.03	1.22	1.36	1.39	1.05	0.827
2	0.800	0.796	1.11	1.05	0.978	
3	0.742	0.812	0.910	0.943	1.34	
**Absorbance / Cutoff[a]**						
**Primate**	**Day 0**	**Day 8**	**Day 18**	**Day 22**	**Day 35**	
1	0.414	0.490	0.550	0.562	0.423	
2	0.323	0.321	0.446	0.424	0.394	
3	0.299	0.328	0.367	0.380	0.539	

[a] Cutoff = Mean Absorbance of Negative Control x 3

Supplementary Table 5. Anti-PS antibody results for CD-1 mice treated with OPLS.

Anti-PS IgG (absorbance at 450 nM)						
**Mouse**	**Vehicle**	**18 mM OPLS**	**45 mM OPLS**	**90 mM OPLS**	**225 mM OPLS**	**450 mM OPLS**
1	0.429	0.297	0.488	0.478	0.270	0.361
2	0.619	0.375	0.306	0.393	0.353	0.318
3	0.318	0.395	0.315	0.524	0.775	0.448
4	0.644	0.297	0.337	0.308	0.725	0.422
5	0.431	0.349	0.591	0.334	1.002	0.606
6	0.377	0.720	0.612	0.276	0.507	0.351
**Absorbance / Cutoff[a]**						
**Mouse**		**18 mM OPLS**	**45 mM OPLS**	**90 mM OPLS**	**225 mM OPLS**	**450 mM OPLS**
1		0.211	0.347	0.339	0.192	0.256
2		0.266	0.217	0.279	0.250	0.226
3		0.280	0.224	0.372	0.550	0.318
4		0.211	0.239	0.219	0.515	0.299
5		0.248	0.419	0.237	0.711	0.430
6		0.511	0.434	0.196	0.360	0.249
**Anti-PS IgM (absorbance at 450 nM)**						
**Mouse**	**Vehicle**	**18 mM OPLS**	**45 mM OPLS**	**90 mM OPLS**	**225 mM OPLS**	**450 mM OPLS**
1	0.753	0.648	0.717	0.357	0.543	0.834
2	0.753	0.744	0.810	0.681	0.5328	0.489
3	0.557	1.116	0.504	0.764	1.727	0.927
4	0.742	0.423	0.765	0.509	0.772	0.785
5	0.865	0.677	0.922	0.864	2.553[b]	0.983
6	0.748	1.517	1.358	0.663	0.953	0.729
**Absorbance / Cutoff[a]**						
**Mouse**		**18 mM OPLS**	**45 mM OPLS**	**90 mM OPLS**	**225 mM OPLS**	**450 mM OPLS**
1		0.293	0.325	0.162	0.246	0.378
2		0.337	0.367	0.309	0.241	0.222
3		0.505	0.228	0.346	0.782	0.420
4		0.192	0.347	0.230	0.350	0.356
5		0.307	0.417	0.391	1.156	0.445
6		0.687	0.615	0.300	0.431	0.330

[a] Cutoff = Mean Absorbance of Vehicle Group x 3
[b] One mouse in the 276 mg/kg OPLS treatment group tested positive for transient PS-reactive IgM antibodies which were resolved in following weeks measurement. The animal that tested positive did not show any other diagnostic features of APS, such as thrombotic events, thrombocytopenia, or prolongation of plasma clotting time (as assessed by activated partial thromboplastin time measurements), suggesting that the development of these antibodies might represent a transient, non-pathological event.

Supplementary Table 6. Serum blood chemistry of CD-1 mice treated with OPLS. Values are presented as mean (SD). Reference ranges were sourced from literature (reference provided in main text).

Parameter	Treatment Group						Reference Range
	Vehicle	OPLS					
		18 mM	45 mM	90 mM	225 mM	450 mM	
Albumin (g/dL)	3.23 (0.3)	3.20 (0.35)	2.8 (0.1)	3.2 (0.26)	3.4 (0.26)	3.7 (1.31)	2.5 - 3.5
Alkaline phosphatase (U/L)	66.0 (27.2)	79.3 (4.5)	57.7 (10.7)	75.3 (39.3)	76.3 (12.0)	99.7 (22.3)	13 - 88
Alanine aminotransferase (U/L)	47.3 (2.5)	31.3 (6.7)	53.3 (7.51)	52.0 (30.3)	44.0 (9.64)	42.0 (14.2)	7 - 227
Amylase (U/L)	1187 (20.6)	1003 (76.4)	1253 (107)	1173 (72.7)	1096 (242)	1150 (69.3)	NR
Total bilirubin (mg/dL)	0.367 (0.06)	0.367 (0.06)	0.33 (0.06)	0.40 (0.0)	0.40 (0.0)	0.37 (0.06)	0.1 - 0.9
Blood urea nitrogen (mg/dL)	19.0 (1.0)	15.3 (0.58)	15.0 (2.0)	17.0 (0.0)	22.7 (2.89)	17 (2.65)	15 - 59
Calcium (mg/dL)	10.1 (0.5)	9.80 (0.36)	9.73 (0.32)	9.63 (0.31)	7.07 (5.26)	9.73 (0.12)	6.8 - 11.9
Phosphate (mg/dL)	7.53 (0.81)	10.2 (1.21)	8.0 (0.44)	7.8 (0.75)	8.77 (0.99)	8.7 (0.53)	6.0 - 11.3
Glucose (mg/dL)	212 (11.7)	220 (8.5)	229 (48)	241 (36.7)	215 (2.89)	193 (2.65)	57 - 197
Sodium (mmol/L)	148 (1.53)	150 (3.61)	148 (1.0)	147 (1.53)	150 (1.15)	150 (1.0)	145 - 175
Potassium (mmol/L)	5.93 (0.51)	6.6 (1.57)	6.6 (1.3)	6.9 (1.28)	6.97 (0.59)	6.87 (1.59)	6.5 - 9.7
Total protein (g/dL)	4.93 (0.23)	4.6 (0.35)	4.63 (0.15)	4.77 (0.21)	5.33 (0.68)	4.67 (0.32)	3.3 - 6.4
Globulin (g/dL)	1.75 (0.07)	1.4 (0.0)	1.87 (0.15)	1.6 (0.17)	1.6 (0.14)	1.6 (0.1)	1.8 - 2.3
